# Characterizing and forecasting the responses of tropical forest leaf phenology to El Nino by machine learning algorithms

**DOI:** 10.1371/journal.pone.0255962

**Published:** 2021-08-26

**Authors:** Taninnuch Lamjiak, Rungnapa Kaewthongrach, Booncharoen Sirinaovakul, Phongthep Hanpattanakit, Amnat Chithaisong, Jumpol Polvichai

**Affiliations:** 1 Department of Computer Engineering, King Mongkut’s University of Technology Thonburi, Bangkok, Thailand; 2 Geo-Informatics and Space Technology Development Agency (GISTDA), Chonburi, Thailand; 3 Department of Environment, Faculty of Environmental Culture and Ecotourism, Srinakharinwirot University, Bangkok, Thailand; 4 The Joint Graduate School of Energy and Environment and Center of Excellence on Energy Technology and Environment, King Mongkut’s University of Technology Thonburi (KMUTT), Bangkok, Thailand; Universidad de Antioquia, COLOMBIA

## Abstract

Climate change and global warming have serious adverse impacts on tropical forests. In particular, climate change may induce changes in leaf phenology. However, in tropical dry forests where tree diversity is high, species responses to climate change differ. The objective of this research is to analyze the impact of climate variability on the leaf phenology in Thailand’s tropical forests. Machine learning approaches were applied to model how leaf phenology in dry dipterocarp forest in Thailand responds to climate variability and El Niño. First, we used a Self-Organizing Map (SOM) to cluster mature leaf phenology at the species level. Then, leaf phenology patterns in each group along with litterfall phenology and climate data were analyzed according to their response time. After that, a Long Short-Term Memory neural network (LSTM) was used to create model to predict leaf phenology in dry dipterocarp forest. The SOM-based clustering was able to classify 92.24% of the individual trees. The result of mapping the clustering data with lag time analysis revealed that each cluster has a different lag time depending on the timing and amount of rainfall. Incorporating the time lags improved the performance of the litterfall prediction model, reducing the average root mean square percent error (RMSPE) from 14.35% to 12.06%. This study should help researchers understand how each species responds to climate change. The litterfall prediction model will be useful for managing dry dipterocarp forest especially with regards to forest fires.

## Introduction

Forest ecosystems are considered as key atmospheric carbon sinks in the global carbon cycle [1]. Recent research has highlighted concerns that forests are adversely affected by climate change, reducing their carbon sink capacity and negatively impacting other ecosystem services [2]. One feature induced by climate change is a shift of weather conditions and patterns, including more frequent extreme weather anomalies [3]. For example, it has been suggested that climate change has increased the intensity and frequency of the El Niño phenomenon [4]. During 2015–2016, one of the strongest El Niño events in the 21st century was reported [5]. This El Niño significantly reduced the amount of rainfall in Southeast Asia and worldwide, and was associated with higher temperatures when compared with the long-term average climate [6]. Related research [7] found the event had significant impacts on forest carbon uptake and species responses which did not appear in the long-term average climate because forests can absorb atmospheric carbon dioxide through photosynthesis.

Phenology is defined as the investigation of temporal patterns in the life cycle of living organisms, correlated with environmental factors during each time period [8]. One important pattern of the forests is the changes in leaf phenology as they respond to various environmental drivers. However, as tropical forest ecosystems are highly diverse, understanding the species-specific responses to such drivers is desirable in order to evaluate the impacts of climate change and implement effective ecosystem management. While some studies have reported on the response of forest to climate change at a community level by using remote sensing data [9–11], there is limited research that studies the relationship between leaf phenology and climate at the species level, especially in tropical dry forest (a subtype of the tropical forest) [12, 13]. Studying the relationship between leaf phenology and climate in tropical dry forests is a challenge because tropical dry forests, although highly diverse, shows similar adaptation patterns across some species. For example, *Sindora siamensis* has the same phenology pattern with *Phyllanthus emblica* [14]. Still, it is well-known that in addition to the photoperiod, seasonal variations in three main factors, namely rainfall, soil moisture and temperature, are responsible for most phenological changes in the tropical dry forests [15]. In the current study, we tried to classify the response patterns of tropical forests based on variations in these three factors.

The objective of this research was to study the impact of climate change on leaf phenology in Thailand’s tropical forests. However, it is difficult to understand the behavior of each species in dry dipterocarp forests (DDFs) because the relationships between leaf phenology and climate change in the tropical dry forest are highly variable and time dependent [14]. In addition to seasonal patterns, there may be stationary changes to mean phenology (i.e., from global warming). In the past, linear regression has been widely applied for analyzing the relationship between climate change and leaf phenology, prediction models for forest management were created based on this analysis. This technique is easy to implement and can illuminate the basic relationships between variables [11–13, 16, 17]. However, the linear regression technique assumes a linear relationship between independent and dependent variables, which is often an oversimplification. A linear model may not be able to capture the complex relationship between climate change variables and leaf phenology. Therefore, this research applied a combination of more powerful techniques to overcome the difficulties in understanding and modeling these phenomena.

One approach to characterize leaf phenology patterns is to use Machine Learning techniques (ML). These techniques are well-known and widely used for analyzing complex data. ML has been applied to modeling and prediction problems in many forestry and ecological studies [18–20]. Self-Organizing Map (SOM) is an ML technique that clusters data based on patterns of similarity. SOM reduces the complexity of high-dimensional data to two dimensions, thus facilitating visualization and analysis [21]. In studying relationship between organisms and their environment, SOM has been effectively applied for clustering numerous organisms [22]. In some forest research, SOM not only helped reduce the complexity of information [23, 24], but was also successful in identifying groups of leaf phenology patterns in DDFs. In fact it provided the best performance when compared with other algorithms [14].

Cross-correlation is another technique that can help model the relationship between climate variables and leaf phenology. Cross-correlation can be used to analyze the temporal patterns relating two sequences of data, in terms of lag time [25], that is, the typical time difference between the change in a controlling and controlled variable. Since leaf phenology and climate measures both vary over time, the cross-correlation technique can be used to clarify the detailed behavior of trees in the tropical dry forest subjected to climate stresses.

As noted above, a linear regression model may not be adequate to capture the relationships between climate variables and leaf phenology, which tend to be non-linear. Long Short-Term Memory (LSTM) is a deep learning technique that is suitable for sequential data [26]. LSTM has been previously applied to analyze the relationship between forests and CO_2_ emissions [27, 28]. LSTM has also been used to monitor and create a phenology prediction model from remote sensing data [29–31].

In this study, the leaf phenology patterns in DDF are grouped using SOM to reduce the complexity of data. Then, the cross-correlation technique is applied to analyze the chronological relationship between the tropical dry forest and the climate at both the community and species levels according to the lag time between leaf phenology changes and litterfall. Lastly, we generate a prediction model for the litterfall data based on the LSTM technique by using the results from the lag time analysis.

## Materials and methods

**Fig 1:**
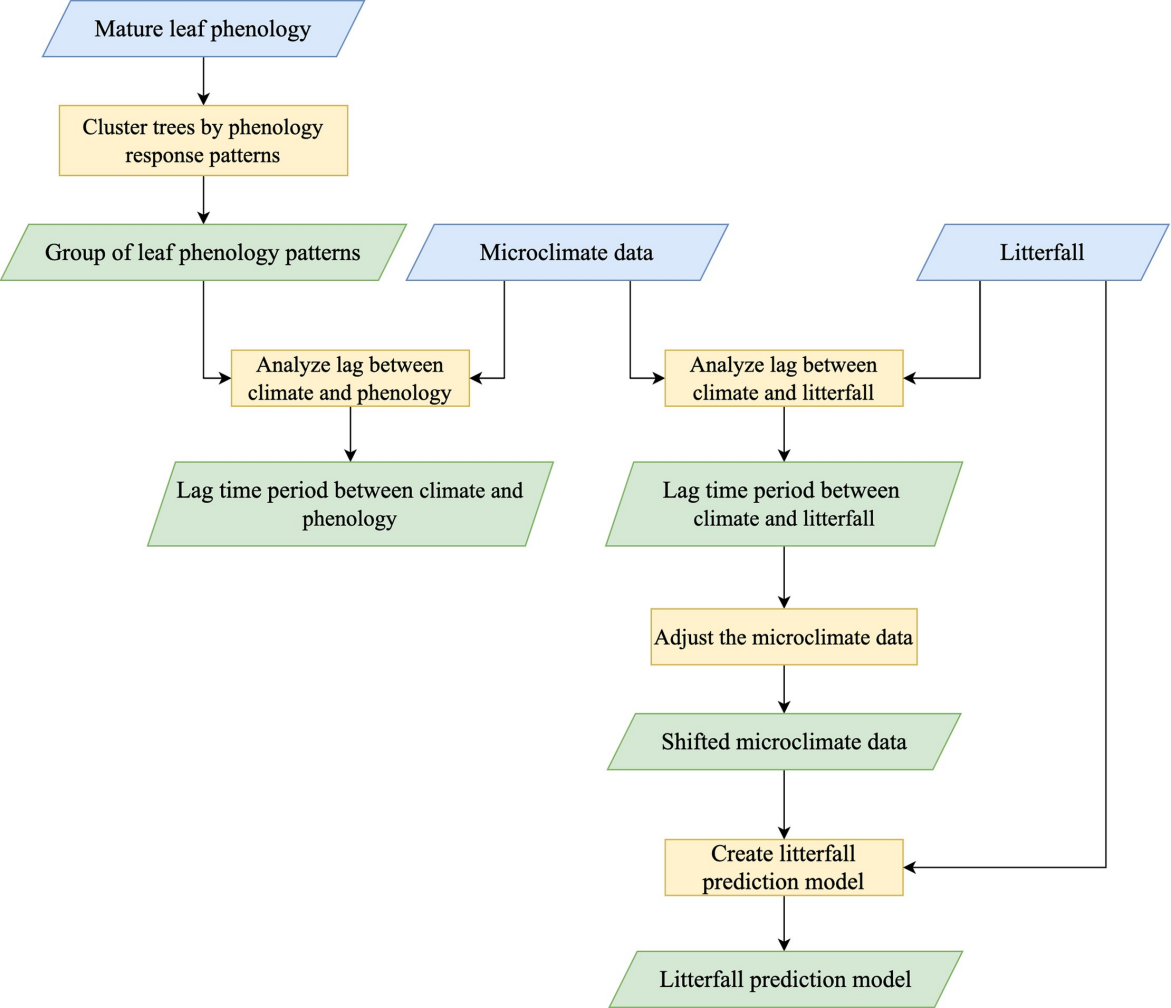
Block diagram of the methodology.

Fig 1 summarizes the methodology used in this research. The process starts with leaf phenology data of each species, that is, measurements of leaf cover at different times. These data are clustered to reduce the diversity of DDF by using the Self-Organizing Map. Then the output, a grouping of trees with similar leaf phenology patterns, is analyzed using cross correlation techniques to get the lag time period between the microclimate data and the phenology patterns. The lag time (also called response time in this paper) shows the dynamics of leaf phenology changes in response to microclimate change. Next, the lag time data are used to adjust the microclimate data by shifting it to correspond to the phenology changes. The time-shifted microclimate data are then used as inputs to create a litterfall prediction model.

### Data

This research utilized data collected from a secondary dry dipterocarp forest (DDF) area in Ratchaburi, Thailand (13° 35’ 13" N: 99° 30’ 4" E, 110 m a.s.l.). There are three main sets of data, explained in more detail below: leaf phenology patterns of each species between 2015–2018; litterfall data between 2009–2018; and microclimate data from the same period. The seasons in this study were divided into dry and wet season following the monsoon rainfall. Normally the dry season starts in November and continues until April, while the wet season runs from May until October [12].

#### Leaf phenology patterns of each species

The mature leaf phenology of 888 trees covering 12 species was manually observed and scored into the 0–4 range over three years (from March 2015 to April 2018). The observed scores were defined based on the percentage of mature leaves on the tree. A score of 0 indicates 0% mature leaves, 1 indicates between 1% and 25% mature leaves, 2 indicates between 26% and 50% mature leaves, 3 indicates between 51 and 75% mature leaves, and 4 indicates from 76 to 100% mature leaves [12, 13]. The data gathering period included the El Niño 2015/2016 phenomenon which was the most severe event of this type since 1950. The 12 observed tree species are included: Litsea glutinosa (Lour.) C.B.Rob, Croton oblongifolius Roxb., Lannea coromandelica (Houtt.) Merr., Erythrophleum succirubrum Gagnep, Dipterocarpus obtusifolius Teijsm. ex Miq, Shorea roxburghii G.Don, Shorea siamensis Miq, Sindora siamensis, Phyllanthus emblica, Shorea obtuse Wall. ex Blume, Xylia xylocarpa (Roxb.) Taub. var. kerrii, Ellipanthus tomentosus.

#### Litterfall data

**Fig 2:**
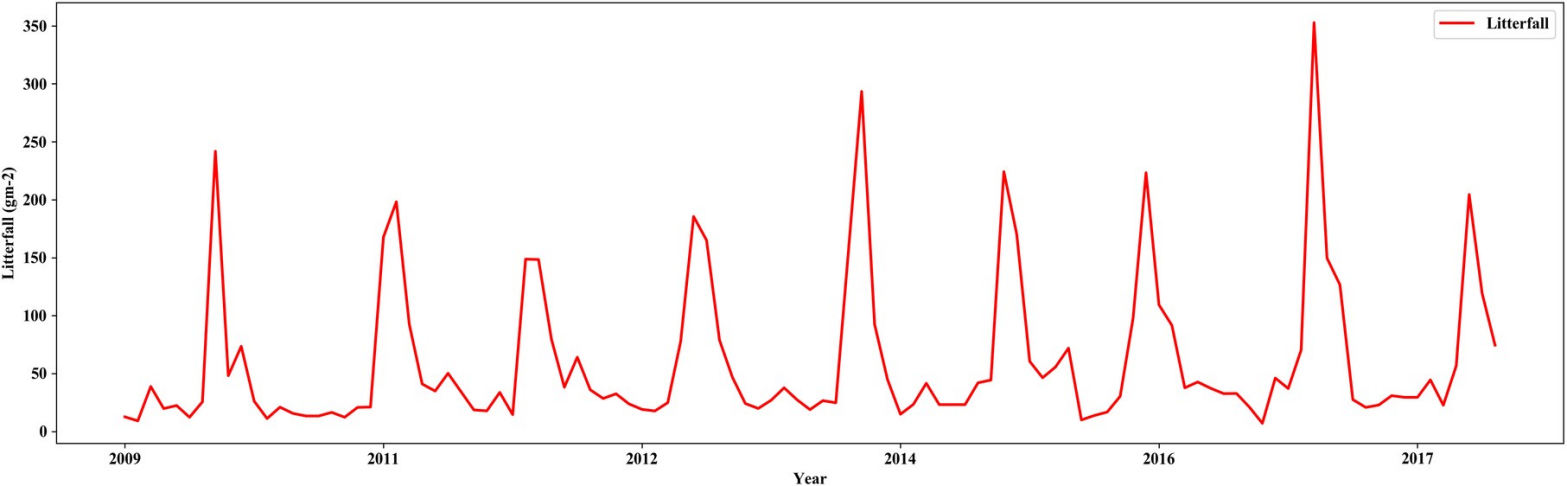
Monthly litterfall data during 2009–2018 used in this study.

Monthly litterfall data were collected for 10 years (from June 2009 to April 2018) by the litter trap technique [32]. The fallen leaves were dried at 75 C for 48 hours and weighed. During this 10-year period, two El Niño events occurred. We used this dataset to create a prediction model to forecast the leaf phenology of the dry dipterocarp forest to guide future forest management. The monthly litterfall data are shown in Fig 2.

#### Microclimate data

Microclimate data related to the leaf phenology were collected from the sensors mounted on a 10-m eddy covariance flux tower. Measured variables included rainfall, soil moisture, Photosynthetically Active Radiation (PAR), Maximum temperature (Tmax), Minimum temperature (Tmin), and Maximum of Vapor Pressure Deficit (VPDmax). A photoperiod dataset was also included in this research, from the geosphere library version 1.5–10 provided in the R language [12, 33]. The microclimate data were compiled for 10 years (from June 2009 to April 2018) and aggregated by monthly averaging. The microclimate data are shown in S1 Fig in S1 Appendix.

### Self-Organizing-Map (SOM)

**Fig 3:**
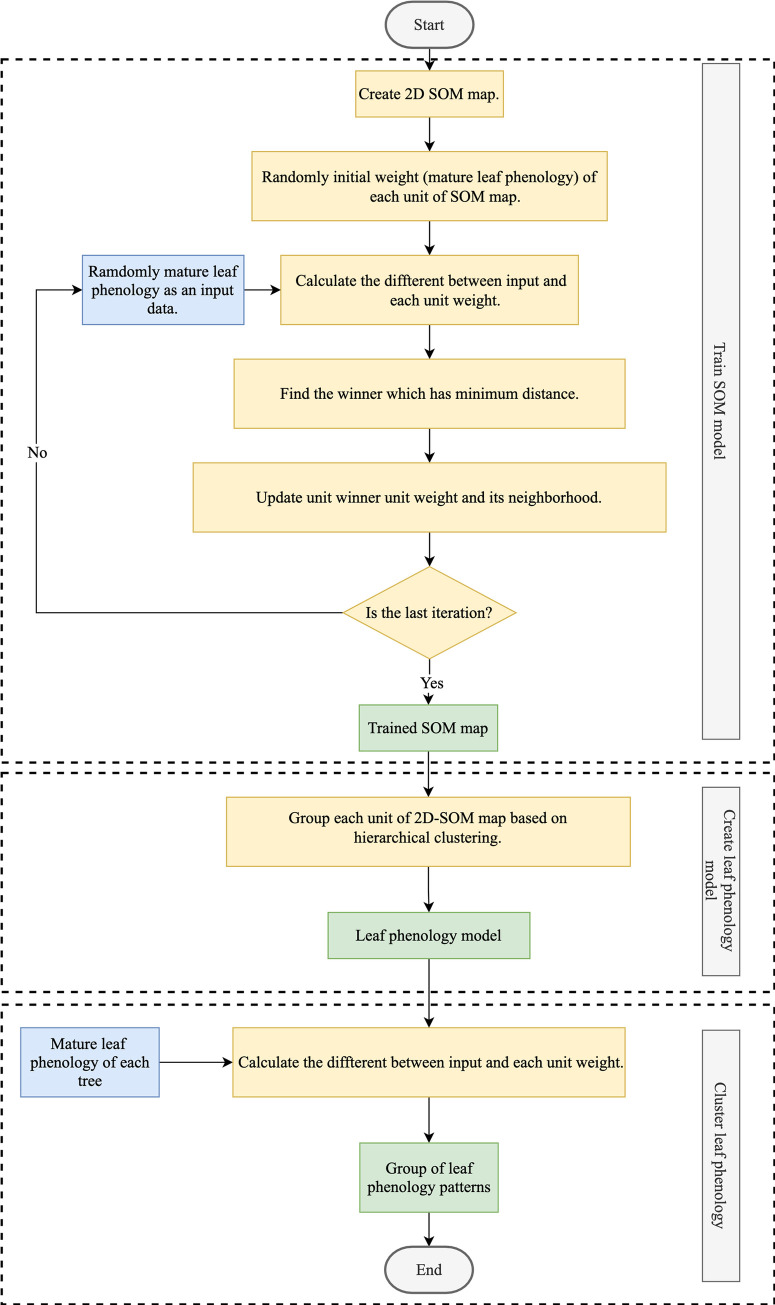
Flowchart illustrating the process of leaf phenology clustering based on SOM.

Although each tree species responds differently to climate, some have similar patterns. To reduce complexity, the tree species were clustered based on their mature leaf phenology by using SOM. Fig 3 shows the process of leaf phenology clustering. The first step is creating the 2-D SOM map. The second step is the grouping each unit of the 2D-SOM map phase. In this step the leaf phenology model is created. Then, the leaf phenology of each tree was clustered from the model.

**Fig 4:**
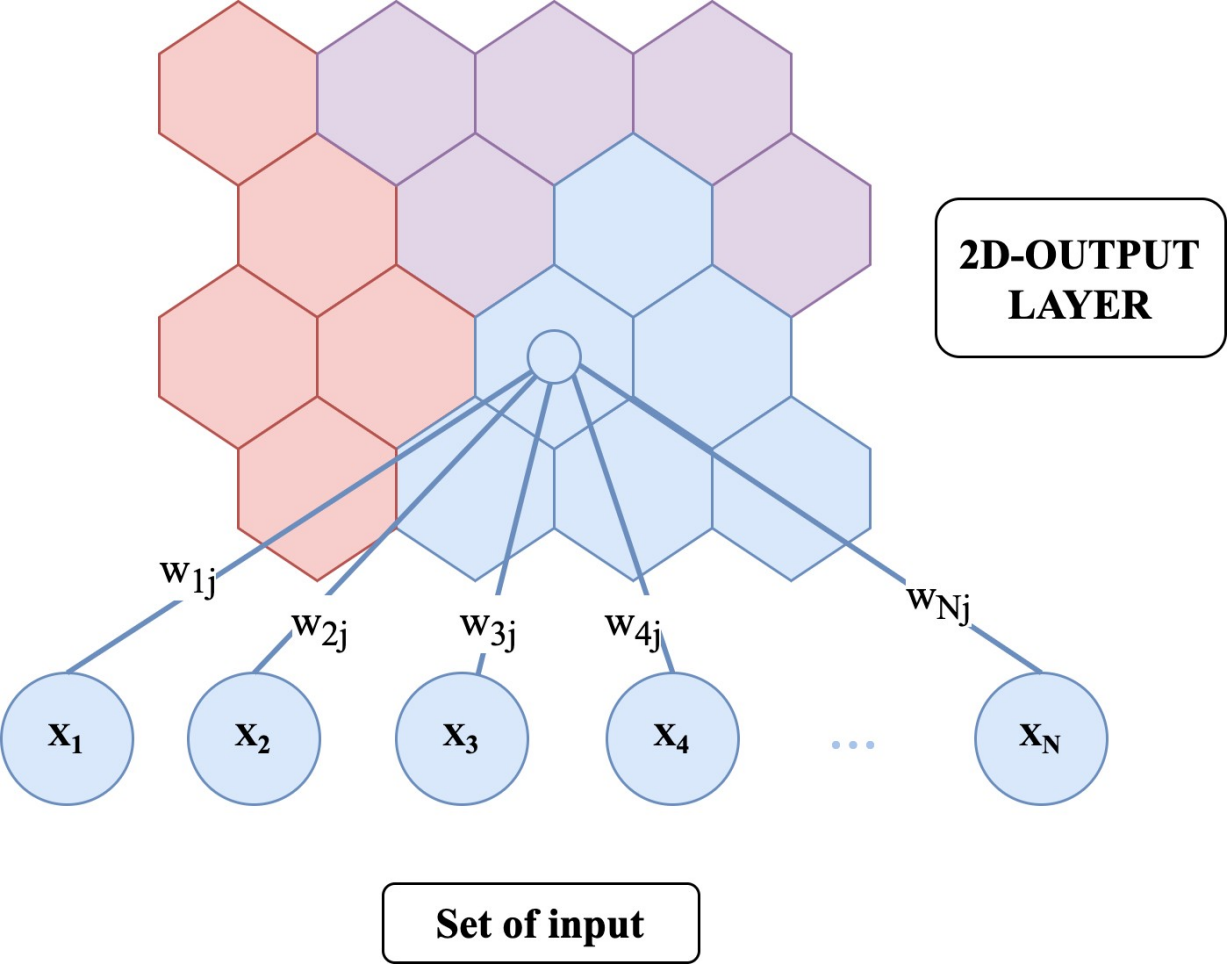
The structure of SOM algorithms.

SOM is an unsupervised machine learning technique which creates a two-dimensional map from complex data. The map represents the similarities or groupings of the input data. SOM contains two layers which are the multiple-dimension input layer (X_1_…X_N)_ and the two-dimensional output layer. The layers are connected with weights (W_1j_….W_Nj_) as shown in Fig 4. In general, SOM has 2 main types of topologies which are grid and hexagon topology. In this research, the patterns of leaf phenology are clustered by using the hexagon topology.

SOM requires the number of desired clusters as an initial parameter. We tried 3 clustering optimization methods to determine the optimal number of clusters: elbow method [34], gap statistic method [35] and average silhouette method [36]. All three techniques indicates that the optimal cluster number is 5.

The training process creates the two-dimensional layer of the SOM map. The size or the number of cell of 2D-map is set to $5*\sqrt{\mathrm{number}\_of\_\mathrm{samples}}$ which is an empirical value used in many studies [37, 38]. The weight value that is used for learning the characteristics of each input is preserved in each unit of the 2D-map. At the beginning of the training process, the weight in each unit is randomly generated. Then an input is selected to train the 2D-SOM map. In our work, the input is the mature leaf phenology pattern of one tree. The Euclidean distance is calculated to represent the difference between weight and input. After calculating the distance between the weight of each unit and input, the node that has a minimum Euclidean distance is selected as the winner to adjust the weight [21].

After training 2D-SOM map, the hierarchical clustering (HC) technique was applied to divide the group of the 2D-SOM map. Each weight unit of the SOM map was used as the input in hierarchical clustering. In this research, the Ward method [39] was used as a criterion to find similarity clusters. The squared Euclidean distance was used to find the dissimilarity of each unit by using Eq (1),

$$d(i,j)=\sum_{k}{\left(x_{ik}-x_{jk}\right)}^{2}$$ (1)

where d is the dissimilarity between unit i and j, x is the unit of the SOM map. After each unit was grouped, the dissimilarity of each group was updated by using Eq (2),

$$d_{(i+j)k}^{new}=\frac{1}{n_{i}+n_{j}+n_{k}}[{(n}_{i}+n_{k})d_{ik}+(n_{j}+n_{k})d_{jk}+n_{k}d_{k}]$$ (2)

where n is the number of units in each cluster.

### Lag time analysis

We analyzed the lag time between the microclimate data and the leaf phenology (species level and community level) to identify which microclimate variables affected both the leaf phenology patterns of each species and the litterfall of the forest. To study the lag time at the species level, five clusters of leaf phenology patterns that were grouped from the clustering process were used as inputs. At the community level, the litterfall data were used to analyze the response time. The cross-correlation technique was applied to understand the lag time of the two independent time series [40]. The the microclimate data and the leaf phenology are the time series data in the current study.

The cross-correlation technique was based on the calculation of the correlation between two shifted sequences, representing two different variables that are related. For example, Table 1 shows shifted rainfall and leaf phenology data gathered for four months. The values of Rainfall and Pattern1 in the columns were shifted monthly ranging from 0 to 4 months to be used as inputs to calculate cross-correlation. The shifted patterns are shown in Table 1. The rows that were Not a Number (NaN) were removed. Therefore, the data used for analysis started from 9/1/2015. Table 2 shows the cross-correlation matrix between four-month shfited data of rainfall and leaf phenology Pattern1 from Table 1. While each row of Rainfall, Reainfall_1mt, Reainfall_2mt, Reainfall_3mt, Reainfall_4mt columns is x variable in Eq (3), each row of Pattern1, Pattern1_1mt, Pattern1_2mt, Pattern1_3mt, Pattern1_4mt is y variable in Eq (3).


$$r=\frac{n(\sum xy)-(\sum x)(\sum y)}{\sqrt{\left[n\sum x^{2}-{\left(\sum x\right)}^{2}][n\sum y^{2}-{\left(\sum y\right)}^{2}\right]}}$$ (3)

**Table 1:** Example of four-month shifted data.

Date m/d/yyyy	Rainfall_0mt	Rainfall_1mt	Rainfall_2mt	Rainfall_3mt	Rainfall_4mt	Pattern1_0mt	Pattern1_1mt	Pattern1_2mt	Pattern1_3mt	Pattern1_4mt
5/1/2015	62.8	NaN	NaN	NaN	NaN	2.9	NaN	NaN	NaN	NaN
6/1/1015	7.9	62.8	NaN	NaN	NaN	3.2	2.9	NaN	NaN	NaN
7/1/2015	35.0	7.9	62.8	NaN	NaN	3.3	3.2	2.9	NaN	NaN
8/1/2015	72.9	35.0	7.9	62.8	NaN	3.6	3.3	3.2	2.9	NaN
9/1/2015	141.4	72.9	35.0	7.9	62.8	4.0	3.6	3.3	3.2	2.9
10/1/2015	177.8	141.4	72.9	35.0	7.9	3.9	4.0	3.6	3.3	3.2
11/1/2015	22.9	177.8	141.4	72.9	35.0	2.2	3.9	4.0	3.6	3.3
12/1/2015	18.6	22.9	177.8	141.4	72.9	0.0	2.2	3.9	4.0	3.6
1/1/2016	18.2	18.6	22.9	177.8	141.4	0.0	0.0	2.2	3.9	4.0
2/1/2016	2.8	18.2	18.6	22.9	177.8	0.0	0.0	0.0	2.2	3.9
3/1/2016	8.0	2.8	18.2	18.6	22.9	0.1	0.0	0.0	0.0	2.2
4/1/2016	0.9	8.0	2.8	18.2	18.6	0.2	0.1	0.0	0.0	0.0

^a^Not a Number (NaN) represents for empty value

^b^ Xmt denotes the number of months shifted where X = [*0*,1,2,3,4]

**Table 2:** The cross-correlation matrix between four-month shfited data of rainfall and pattern1.

	Rainfall_0mt	Rainfall_1mt	Rainfall_2mt	Rainfall_3mt	Rainfall_4mt
**Pattern1_0mt**	0.69	0.51	0.17	-0.19	-0.37
**Pattern1_1mt**	0.53	0.68	0.49	0.13	-0.20
**Pattern1_2mt**	0.31	0.52	0.66	0.45	0.10
**Pattern1_3mt**	0.12	0.31	0.51	0.62	0.42
**Pattern1_4mt**	-0.09	0.11	0.29	0.48	0.60

^a^Xmt denotes the number of months shifted where X = [0,1,2,3,4]

### Long Short-Term Memory prediction model

Deep learning Long Short-Term Memory (LSTM) was used to create a model to predict litterfall from microclimate data. Models trained using non-shifted and shifted data were compared, in order to examine whether considering the lag time between the microclimate data and the leaf phenology would help improve the performance of the prediction model.

**Fig 5:**
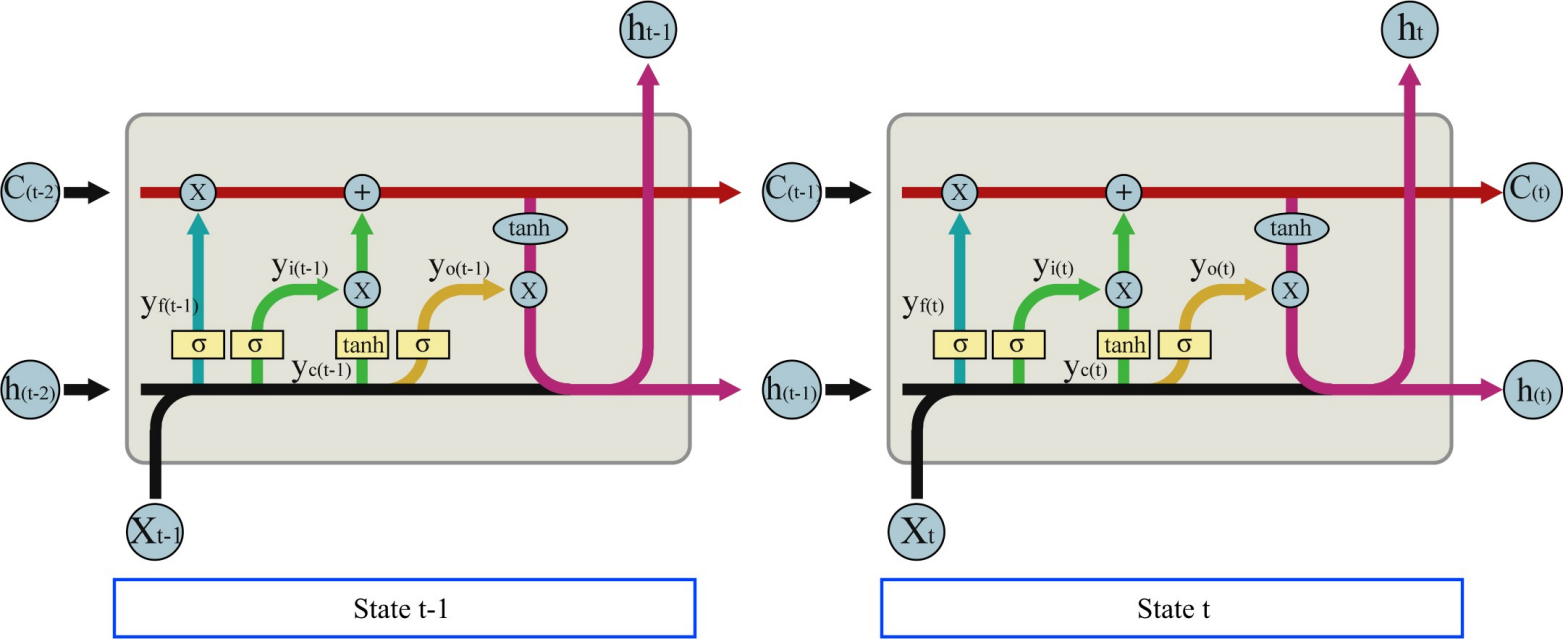
LSTM structure.

Fig 5 shows the structure of the LSTM model. C represents a cell state. Cell state is the core of LSTM. In theory, cell state contains the relevant information throughout the processing of the sequence. X is sequence input, while h represents for output of each state which is an amount of litterfall. There are three inputs. The first input of the present cell is X(t) that contains microclimate data at time (t) and litterfall data at time (t-1), second, the output from previous cell(t-1) is h(t-1) that is the predicted amount of litterfall, and the last cell state from the previous cell(t-1) is C(t-1). The outputs of current cell(t) in LSTM are cell state C(t) and output h(t). The cell state C(t) serves to decide whether the incoming information should be remembered or not. C(t) can be calculated using Eq (4),

$$C(0)=0,C(t)=y_{f}(t)C(t-1)+y_{i}(t)tanh\;(y_{c}(t))$$ (4)

where y_f_(t) is the output of the forget gate, y_i_(t) is the output of the input gate and y_c_(t) is the output of the output gate which are calculated using Eqs (5), (6) and (7), respectively.


$$y_{f}(t)=\sigma ((\sum_{m}W_{hfm}*h_{m}(t-1))+(\sum_{m}W_{Xfm}*X_{m}(t))+b_{f})$$ (5)

$$y_{i}(t)=\sigma ((\sum_{m}W_{him}*h_{m}(t-1))+(\sum_{m}W_{Xim}*X_{m}(t))+b_{i})$$ (6)

$$y_{c}(t)=\sum_{m}W_{hcm}*h_{m}(t-1)+\sum_{m}W_{Xcm}*X_{m}(t)+b_{c}$$ (7)

While σ is the sigmoid function, W_hm_ and W_Xm_ are the learning weight of input h(t-1) and input X(t) in the forget gate, and b_i_ is the bias value. The output h(t) of each cell state can be calculated using Eq (8),

$$h(t)=y_{o}*tanh(C(t))$$ (8)

y_o_(t) is the output of the output gate which can be calculated using Eq (9),

$$y_{o}(t)=\sigma ((\sum_{m}W_{hom}*h_{m}(t-1))+(\sum_{m}W_{Xom}*X_{m}(t))+b_{o})$$ (9)

### Experimental settings and evaluation method

#### Evaluating the performance of the clustering algorithms

The performance of SOM algorithm was compared with the output from KMeans, Hierarchical Clustering (HC) and Gaussian Mixture Model (GMM). To validate the performance of the clustering algorithms, the S_Dbw_ validity index was used [41]. This metric simultaneously evaluates 5 characteristics: monotonicity, noise, density, sub-clusters, and skewed distributions. S_Dbw_ takes the scattering and density of information in each cluster to measure the inter-cluster separation. A smaller index indicates better clustering results. The S_Dbw_ validity index is calculated by using Eq (10),

$$S_{Dbw}=SCATT+DENS\_BW$$ (10)

where SCATT represents the average scattering for clusters that is calculated based on Eq (11).


$$SCATT=\frac{\sum_{i=1}^{c}\left\|\sigma (v_{i})\right\|}{c\|\sigma (S)\|}$$ (11)

While σ(v_i_) is the standard deviation of Euclidean distance of the data in each cluster, σ(S) which is the standard deviation of Euclidean distance of all data is used as a normalization factor to constrain the range of SCATT value. The variable *c* represents the number of clusters. A smaller value of SCATT indicates better results since it is desirable that each cluster has low variance. DENS_BW is inter-cluster density that is defined by Eq (12),

$$DENS_{BW}=\frac{\sum_{i=1}^{c}\left(\sum_{\begin{array}{c} j=1\\ j\neq 1 \end{array}}^{c}\frac{dense(u_{ij})}{max(dens(v_{i},dens(v_{j})))}\right)}{c(c-1)}$$ (12)

where dens(v_i_) is the density of each cluster and dense(u_ij_) is the density between cluster i and j. The density of each cluster and between two clusters can be calculated from Eq (13),

$$dense(v)=\sum_{i=1}^{n_{ij}}f(X_{i},v)$$ (13)

where n_ij_ is a number of data in each cluster. v is the centroid of dense(v_i_) and dense(u_ij_). Eq (14) represents the amount of data in the neighborhood area that can find from function f(Xi,v).


$$f(x,y)=\left\{ \begin{array}{l} 0,\;if\;d(x,v)>stdev\\ 1,\;otherwise \end{array}\right.$$ (14)

Where d(x,v) is the Euclidean distance between each data and the centroid and stdev is the average standard deviation of data point that used as a neighborhood area. stdev can be calculated from Eq (15),

$$stdev=\frac{\sqrt{\sum_{i=1}^{c}\left\|\sigma (v_{i})\right\|}}{c}$$ (15)

#### Evaluating the performance of the prediction model

The LSTM algorithm used in this research was implemented in Python using Keras Application Programming Interface (API) [42]. The performance of the LSTM technique was compared to state-of-the-art prediction algorithms including Linear Regression [43], Regression Tree [44], Artificial Neural Network (ANN) algorithms [45], and ARIMAX [46]. To test whether the response time analysis affected the predictive modeling, the performance of the model trained with the general input data and that of the model trained with the shifted input data (the results from the lag time analysis process) were compared.

As the data used in this research are sequential data, the root mean square percent error (RMSPE) was used to evaluate the performance of the experimental technique [47]. RMSPE can be calculated using Eq (16),

$$RMSPE=\sqrt{\frac{\sum_{i=1}^{n}{\left({Predicted}_{i}-{Actual}_{i}\right)}^{2}}{n}*100\%}$$ (16)

where n represents the number of samples of testing data. To prevent model overfitting, 5-fold cross-validation [48] was used and the models were run 10 times when testing the performance of the algorithms.

### Experimental results

#### Clustering leaf phenology patterns

The clustering results of each algorithm are shown in Table 3. Table 3 shows that the number of trees in each species that were included in some cluster in Self-Organizing Map (SOM) is higher than other algorithms which means SOM does a better job than other algorithms of covering the full data set. The S_Dbw_ index for each algorithm is shown in Table 4. It is obvious that SOM provided the best index, while GMM provided the worst index. The indices for SOM, KMeans, and Hieratical Clustering (HC) are fairly similar to each other. This may be because Euclidean distance is used to measure the dissimilarity of SOM, KMeans, and HC, but Gaussian Mixture Model (GMM) clustering is based on probability. Since, SOM provided better performance than other algorithms, we concluded that the leaf characteristics of each species should be grouped using SOM. From Table 3, the clustering result of SOM and HC are similar. In SOM and HC, while L. coromandelica was clustered into the 1^st^ group, D. obtusifolius and E. tomentosus were clustered into 2^nd^ group. The third group produced by the clustering contained most species in study area, including L. glutinosa, C. oblongifolius, S. siamensis, Sindora siamensis, P. emblica, S. obtuse and X. xylocarpa. E. succirubrum and S. roxburghii was clustered into the 4^th^ and 5^th^ group respectively.

**Table 3:** Clustering results of 12 species obtained by considering each tree based on SOM compared with other algorithms [14].

Species Name	Self-Organizing Map		Hieratical Clustering		K-Mean		Gaussian Mixture Model		R. Kaewthongrach et. al [12]
	Group number	Number of trees (%)	Group number	Number of trees (%)	Group number	Number of trees (%)	Group number	Number of trees (%)	Group number
*L*. *coromandelica*	1	100%	1	100%	1	100%	1	100%	1
*D*. *obtusifolius*	2	97.14%	2	95.71%	2	94.28%	2	91.30%	2
*E*. *tomentosus*	2	100%	2	94.44%	2	86.96%	2	98.57%	2
*L*. *glutinosa*	3	75%	3	75%	3	100%	5	86.96%	5
*C*. *oblongifolius*	3	78.26%	3	73.91%	3	50%	2	75%	5
*Shorea siamensis*	3	95.31%	3	89.06%	5	54.34%	3	58.92%	3
*Sindora siamensis*	3	100%	3	100%	3	100%	2	100%	3
*P*. *emblica*	3	100%	3	100%	5	87.50%	3	46.88%	3
*S*. *obtuse*	3	76.47%	3	70.59%	5	89.06%	2	100%	5
*X*. *xylocarpa*	3	100%	3	100%	5	100%	3	80%	3
*E*. *succirubrum*	4	87.50%	4	100%	4	52.94%	4	62.50%	4
*S*. *roxburghii*	5	97.14%	5	86.95%	2	100%	2	50%	5
	Average number of trees (%)	**92.24%**	Average number of trees (%)	90.47%	Average number of trees (%)	84.59%	Average number of trees(%)	78.08%	

**Table 4:** The results of internal validation by using the S_Dbw_ validity index [14].

Clustering Algorithms	S_Dbw_ index
Self-organizing map	**0.772**
Kmean	0.780
Hieratical Clustering	0.787
Gaussian Mixture Model	0.829

**Fig 6:**
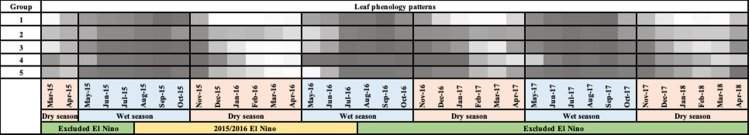
The five main leaf phenology patterns clustered by SOM [14]. The shades of grey represent for the average amount of mature leaf phenology. The darker greys represent more mature leaves.

As shown in Fig 6, the leaf phenology patterns of 12 species were clustered in to 5 groups and can be described as follows:

- Group 1: Long completely deciduous period (≈5 months) both during the El Niño phenomenon and in normal years.
- Group 2: Incompletely deciduous both during the El Niño phenomenon and in normal years.
- Group 3: Short completely deciduous period (≈1 months) both during the El Niño phenomenon and in normal years but with a longer deciduous period (≈6 months) in the El Niño phenomenon.
- Group 4: Incompletely deciduous in the usual season. By contrast, in the El Niño phenomenon, the leaf phenology is completely deciduous for a short period (≈3 months).
- Group 5: Incompletely deciduous in the usual season. By contrast, in the El Niño phenomenon, leaf phenology is completely deciduous for a long period (≈6 months).

#### Lag time analysis between the microclimate data and the leaf phenology covering severe drought versus normal seasons

**Fig 7:**
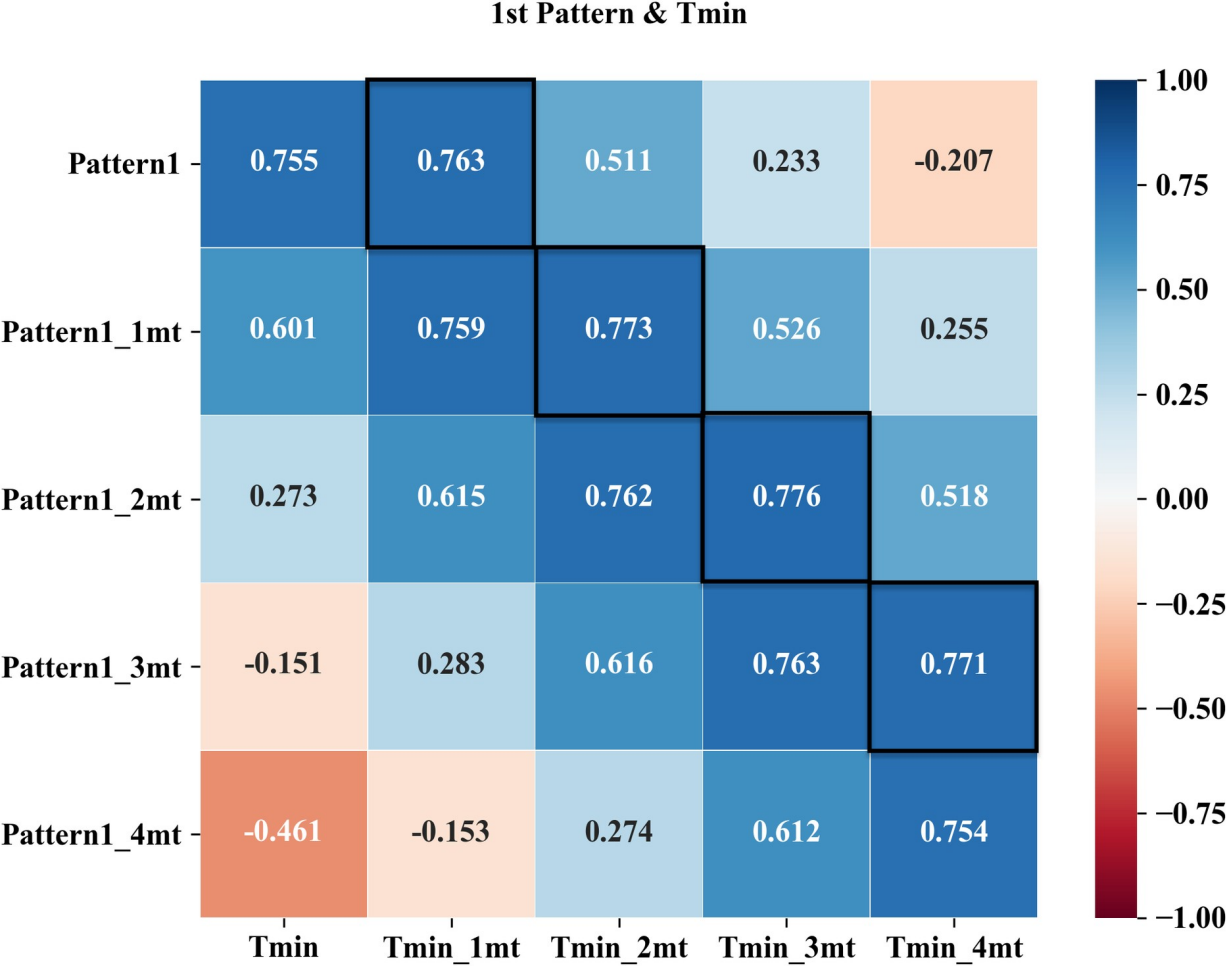
Heatmap of cross-correlation between the 1st group of leaf phenology patterns and the minimum temperature. Number in each cell is a correlation value between row and column. The color in each cell represents the correlation value, range between -1 to 1. The dark blue color represents the highest positive correlation (1), White represents no correlation (0), and Dark red represents the highest negative correlation (-1). The threshold color column shown in the righthand side represent the level of correlation value. The cells that have black border represent the highest correlation between leaf phenology and minimum temperature.

In general, leaf phenology is highly sensitive to climate factors and climate factors lead the phenological change. However, the phenology responds to various climate factors with apparent and different lag time [49]. Considering the lag time effects is quite important for better understanding of vegetation-climate interaction and development of the models [49–52]. The results of lag time analysis between leaf phenology patterns and microclimate data are visualized as a heatmap. Fig 7 provides an example. The figure shows the relationship between the leaf phenology of *L*. *coromandelica* which was clustered into group 1 and the minimum temperature. The heat map shows that *L*. *coromandelica* has a high correlation after the minimum temperature lasting for one month. Therefore, we are able to conclude that *L*. *coromandelica* adapted themselves following the one-month minimum temperature. The detailed results of the lag time analysis are shown in S2 Fig in S2 Appendix.

Table 5 shows the time relationship between each group of the leaf phenology patterns and the microclimate data derived from the cross-correlation technique. *L*. *coromandelica* was clustered into the 1st group and *D*. *obtusifolius* and *E*. *tomentosus* were clustered into the 2nd group. These species do not adapt their leaf phenology to follow rainfall and soil moisture. However, they adapt themselves to follow Tmin and photoperiod in different periods. On the other hand, the tree species that were clustered into Group 3 (*L*. *glutinosa*, *C*. *oblongifolius*, *Shorea siamensis*, *Sindora siamensis*, *P*. *emblica*, *S*. *obtuse*, *X*. *xylocarpa*), Group 4 (*E*. *succirubrum*) and Group 5 (*S*. *roxburghii*) adapt themselves to follow rainfall and soil moisture. As most of the dominant species in the study area [12] were clustered into Group 3, soil moisture and rainfall can be considered as the key driving factors of leaf phenology patterns in this forest ecosystem. The relationship between the litterfall data at the community level and the microclimate resembles the leaf phenology pattern in Group 3. Furthermore, the results show that relationships between the leaf phenology in DDF and Tmax, VPD, and PAR are negative. That is, mature leaf phenology increases when Tmax, VPD, and PAR decrease and vice versa. On the other hand, while the mature leaf phenology decrease, Tmax, VPD and PAR are increase.

**Table 5:** The lag time between each leaf phenology pattern and microclimate.

	Rainfall	Soil moisture	Tmin	Photoperiod	Tmax	VPDmax	PAR
**Litterfall**	+1	0	+2	+3	-2	-1	-2
**1** ^**st**^ **Group**	0	0 or -1	+1	+2	-2	-2	-2
**2** ^**nd**^ **Group**	0	0 or -1	+1	+3	0	0	0 or -1
**3** ^**rd**^ **Group**	+1	0	+1	+3	-1 or -2	-1	-1
**4** ^**th**^ **Group**	+1	0 or +1	+1	+3	-1	0	-1
**5** ^**th**^ **Group**	+1	0 or +1	+2	+3	-1	0	0

“+” means the changes of the leaf phenology pattern are detected after the changes of microclimate variables, “-”means the changes of the leaf phenology pattern are detected before the changes of microclimate variables. Number 1–3 represents the number of months lag between the leaf phenology change and the microclimate change. Number 0 mean leaf phenology patterns change at the same time as with microclimate.

After comparing the results from Table 5 with the average of the leaf phenology pattern in DDF in Fig 6, we found that the result from Table 5 supports the clustering of leaf phenology at the species level based on SOM algorithms. The results from Fig 6 and Table 5 show that the leaf phenology patterns in Group 1 and Group 2 do not change in the severe drought period because neither group is sensitive to rainfall nor soil moisture, but they both are sensitive to Tmin and photoperiod. As the duration of day time in Thailand is not significantly different in each season even during the El Niño phenomenon, the leaf phenology the first group and the second group do not change during the El Niño phenomenon. On the other hand, the leaf phenology pattern in Groups 3, 4 and 5 are sensitive to rain and soil moisture in different periods. Therefore, the El Niño occurrence in 2015/2016 caused their leaf phenology to become completely deciduous for a longer period as a result of the delay of rainfall. Moreover, the result from the Table 5 shows that the species that fall into the incompletely deciduous leaf phenology pattern include Group 2, Group 4, and Group 5, which adapt themselves simultaneously with Tmax, VPDmax and PAR. Nevertheless, the result shows that the phenology of *E*. *succirubrum* in Group 4 and *S*. *roxburghii* in Group 5 are quite similar to each other. Moreover, the periods during which *E*. *succirubrum* and *S*. *roxburghii* respond to the microclimate are very similar. Hence, the leaf phenology of *E*. *succirubrum* and *S*. *roxburghii* can be considered as falling into the same group.

#### Litterfall prediction model with machine learning techniques

Litterfall phenology was predicted by using microclimate data. LSTM was applied to create a prediction model. The RMSPE was employed to evaluate the performance of the prediction model. Table 6 shows the performance results of the litterfall phenology prediction model based on LSTM compared with other state-of-the-art approaches including ANN, ARIMAX, Regression Tree, and Linear Regression. Table 6 also compares the performance results with two training data sets, the original dataset and the shifted dataset. The shifted dataset was derived from the lag time analysis process. As can be seen from columns 3 and 4 in Table 6, most of the models that used shifted parameters based on the lag period from the cross-correlation analysis approach as input factors produce better performance than those that used the original parameters. Only the result from ARIMAX produce a lower average and minimum RMSPE using the original parameters than shifted parameters as shown in row 3 in Table 6. Linear regression, which was the least accurate approach based on RMSPE, was slightly better with the original parameters than the shifted parameters as shown in row 5 in Table 6. As indicated in the underlined RMSPE in columns 3 and 4, LSTM provides better results than other approaches for almost every metric except the minimum RMSPE, with both the original and the shifted parameters. Furthermore, the values of the mean, the minimum, and the maximum of RMSPE are not markedly different. The best result of the litterfall prediction model is from the LSTM approach using shifted parameters as an input factor which has a mean RMSPE equal to 12.06%. This is an improvement of more than 2% over the non-shifted data, which produces a mean RMSPE of 14.35%.

**Table 6:** The performance of litter fall prediction models as evaluated by RMSPE.

Algorithms	Stat	Original Parameters	Shifted Parameters
**LSTM**	**Mean**	14.35	**12.06**
	**Min**	12.59	**11.50**
	**Max**	15.32	**12.51**
	**SD**	1.03	**0.40**
**ANN**	**Mean**	17.04	**16.15**
	**Min**	16.22	**15.06**
	**Max**	18.77	**18.01**
	**SD**	**1.04**	1.33
**ARIMAX**	**Mean**	**23.26**	24.79
	**Min**	**17.01**	18.56
	**Max**	30.49	**29.48**
	**SD**	5.07	**4.56**
**Regression Tree**	**Mean**	23.32	**22.56**
	**Min**	17.63	**9.69**
	**Max**	**29.01**	30.72
	**SD**	**4.58**	8.08
**Linear Regression**	**Mean**	16.74	**16.67**
	**Min**	10.74	**10.46**
	**Max**	**21.08**	21.21
	**SD**	**3.85**	4.28

## Discussion

Understanding of the relationship between the behavior of each tree species and climate in the tropical dry forest is one of the challenges in forest ecological studies. There are many tree species in the tropical dry forest that have different characteristic phenologies, meaning that they respond differently to the climate. In this research, the behavior of 12 species in DDF with respect to microclimate was investigated by applying a machine learning approach that is suitable to predict sequence data. Due to the variety of characteristics of leaf phenology in the observed data, SOM was applied to reduce the complexity from 12 species into 5 main groups. The experimental results have shown that SOM provides the best clustering performance when compared with other state-of-the-art algorithms. In addition, the results in this research were also compared with the results from previous research [12] as shown in Table 3, indicate that groups 1, 2 and 4 in the last column of Table 3 were clustered in the same way. However, group 3 and 5 were clustered differently. *L*. *glutinosa*, *C*. *oblongifolius*, and *S*. *obtuse* in group 3 in this research were clustered into group 5 in [12]. The reason that these 3 species were grouped into group 5 in the last column of Table 3 is due to the fact that the average phenology of 3 species, which was used as clustering input in the last column of Table 3, is more similar to group 5 than group 3. However, when considering the information of every tree, the number of trees that were clustered in group 3 is 75%, 78.26%, and 76.47% respectively in the SOM algorithm. These results show that the clustering of leaf characteristics for each species should consider each tree individually. This permits extraction of more specific and informative results than the average phenology data.

The clustering results from SOM were used as input data for lag time analysis to improve our understanding of response of species groups to climate drivers. In this part, litterfall data was used to study how the DDF in the study area responds to the microclimate at the community level. The results show that the cross-correlation approach can explain how different species adapt themselves to microclimate. Even though the correlation value between rainfall and leaf phenology is not high, most of the tree species including *L*. *glutinosa*, *C*. *oblongifolius*, *S*. *siamensis*, *Sindora siamensis*, *P*. *emblica*, *S*. *obtuse*, *X*. *xylocarpa*, *E*. *succirubrum*, *S*. *roxburghii* adapt themselves to follow rainfall and soil moisture. A possible explanation for the low correlation between rainfall and leaf phenology is that rainfall data are highly variable while change in leaf phenology is a slow process.

Because this study used the information covering both normal and severe drought seasons, it can examine different drought-related patterns in each tree species. The results produced five different groups of tree species. For two groups, a precipitation deficit apparently does not affect their behavior. However, in both severe drought and general dry season, one group is completely deciduous with a long duration and another group is incompletely deciduous. The next group is completely deciduous for approximately one month in usual dry season but has longer period, up to four months, during a severe drought event. Furthermore, in the normal dry season the remaining two groups are incompletely deciduous but completely deciduous during the El Niño phenomenon. The former group has deciduous period in El Niño phenomenon shorter than the latter group.

The cross-correlation approach describes how the leaf phenology patterns differ from each other. The results support the hypothesis of Hernández et al. and Kaewthongrach et al. [12, 15] which states that most phenological changes in dry tropical forests are caused by three leading factors: seasonal variation in rainfall, seasonal variation in soil moisture and temperature and photoperiod. In our results, the leaf phenology of the first and second groups are affected by minimum temperature and day length more than rainfall which corresponds to the study by [53, 54]. In addition, because most species in the study area are in the third group, which is affected by El Niño, the amount of litterfall was increased during El Niño which created severe drought conditions. However, the experimental results have not been able to clearly explain the behavior of trees at the species level because there may be many parameters in the ecosystem that affect the phenology of trees. In this research, we present the facts obtained from experimental results that explain the behavior of trees using mathematical techniques, based on the collected leaf phenology and microclimate data, and these points need further study into the details.

The results from lag time analysis tended to improve the performance of the prediction model. The LSTM approach which provides the best solution overall, improves its mean RMSPE value from 14.35% to 12.06% when time-shifted data are used as input.

Our research enables us to gain an insight into the behavior of each species cluster in DDF. The results from our study have some implications. Litterfall seasonality is one of the characteristics that represents the behavior of trees at the community level. During the dry period, the litterfall on the forest floor can serve as fuel for forest fires. Therefore, the litterfall prediction model can be used for wildfire pevention and management as well as for afforestation and reforestation for sustainable land management.

## Conclusions

The goal of this research was to better understand the behavior of each tree species in the secondary dry dipterocarp forest in response to climate in both usual season and El Nino phenomenon. Recognizing the biodiversity of DDF, we used SOM to cluster 12 species of trees from DDF into groups based on leaf phenology patterns. Then, we studied the characteristics of leaf phenology patterns in response to microclimate. Microclimate variables included rainfall, soil moisture, photosynthetically active radiation (PAR), maximum temperature (Tmax), minimum temperature (Tmin), and maximum of Vapor pressure deficit (VPDmax). We analyze the lag time between microclimate data, leaf phenology patterns and litterfall data by a cross-correlation approach to get response time data. Then, the response time data is used as an input of LSTM to create the prediction model that will predict the amount of litterfall according to the microclimate data.

The results show that SOM is the most suitable clustering appraoch for leaf phenology data compared to other state-of-the-art approaches as it provides the lowest S_Dbw_ index and the highest coverage of individual data points. The 12 species in DDF were clustered into 5 main groups according to their leaf phenology patterns which respond differently to microclimate data. The results also showed that leaf phenology patterns of the dominant species in DDF, group 3, was affected by the El Niño phenomenon. However, there are some species in groups 1 and 2 whose phenology is related to day length and minimum temperature rather than scant rainfall caused by El Niño phenomenon.

Furthermore, we found that an LSTM-based model was effective for predicting litterfall based on microclimate data. Since the leaf phenology and microclimate data are related in time, incorporating the response lag into the litterfall prediction model improved that model’s performance.

## Supporting information

S1 Appendix Microclimate data. (PDF)

S2 Appendix The detail results of lag time analysis. (PDF)
