# Supplementary material for: Characterizing and forecasting the responses of tropical forest leaf phenology to El Nino by machine learning algorithms
Source: PLoS One. 2021 Aug 26;16(8):e0255962. doi: 10.1371/journal.pone.0255962 (PMC8389403; doi:10.1371/journal.pone.0255962)
Supplement: S1 Appendix — (PDF) [file pone.0255962.s001.pdf]

S1 Appendix. Microclimate data

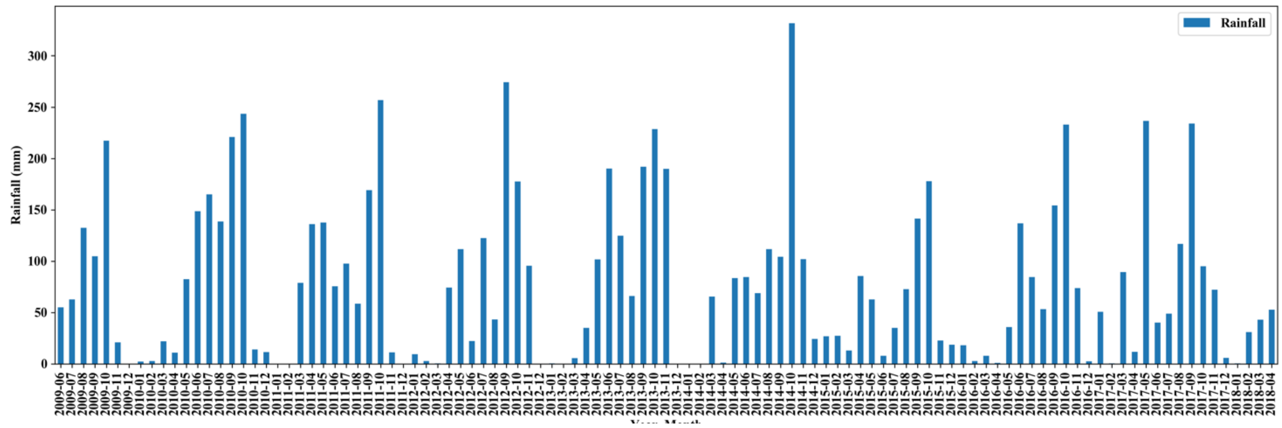

S1 Fig1. Ten years of monthly rainfall.

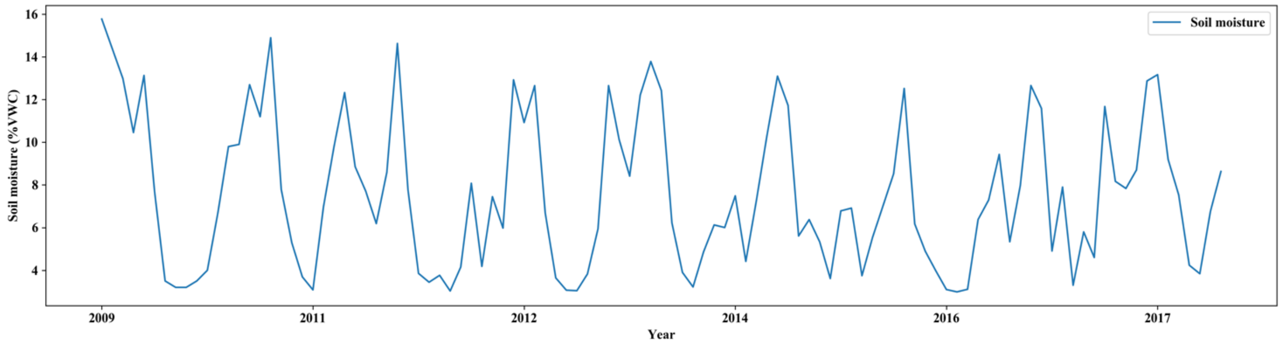

S1 Fig2. Ten years of monthly soil moisture.

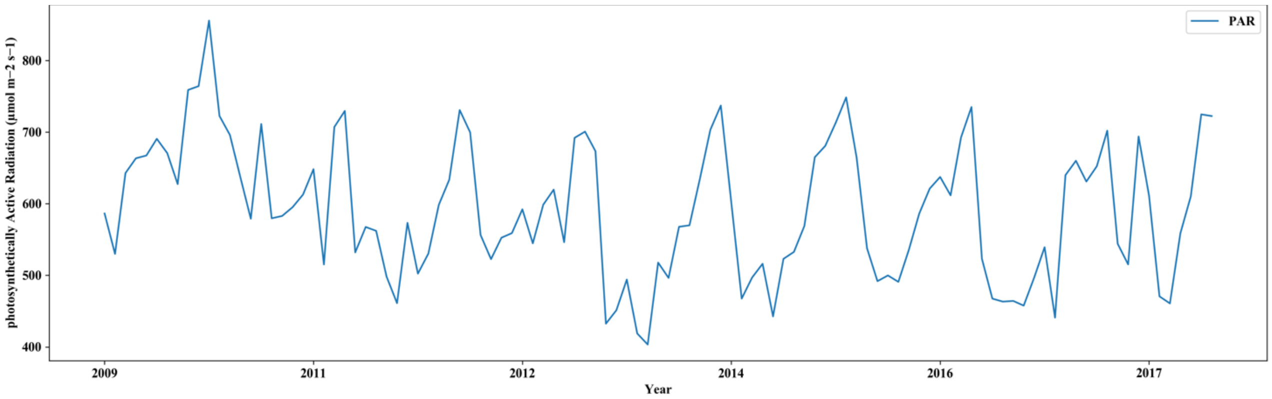

S1 Fig3. Ten years of monthly photosynthetically active radiation.

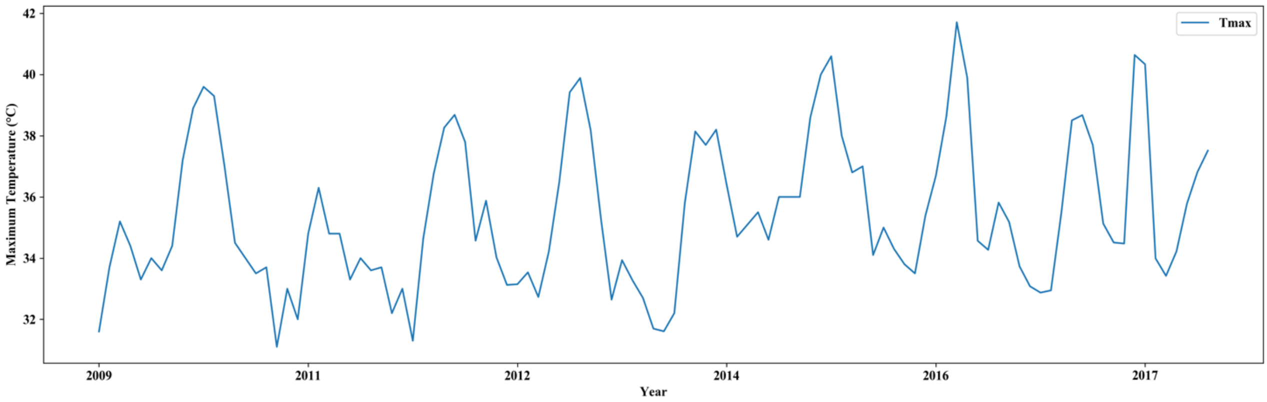

S1 Fig4. Ten years of monthly maximum temperature.

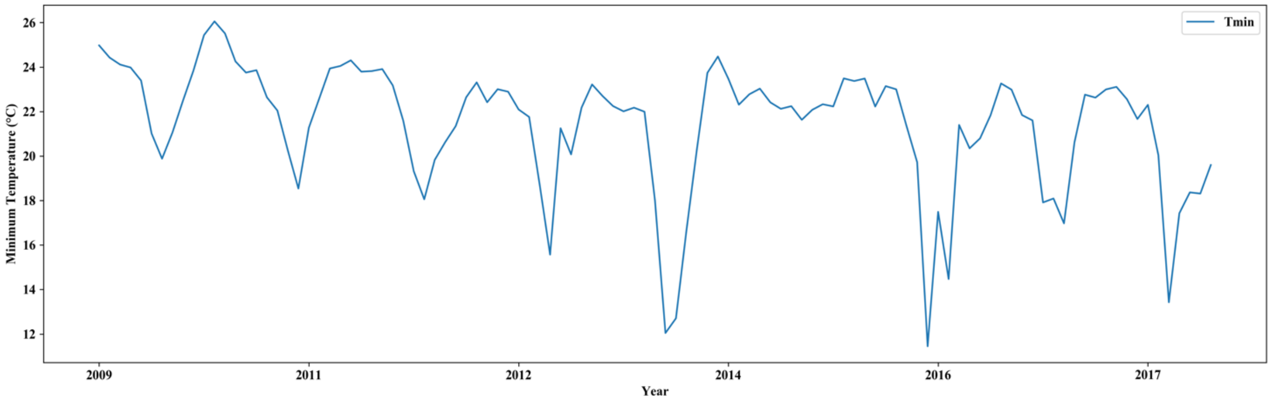

S1 Fig5. Ten years of monthly minimum temperature.

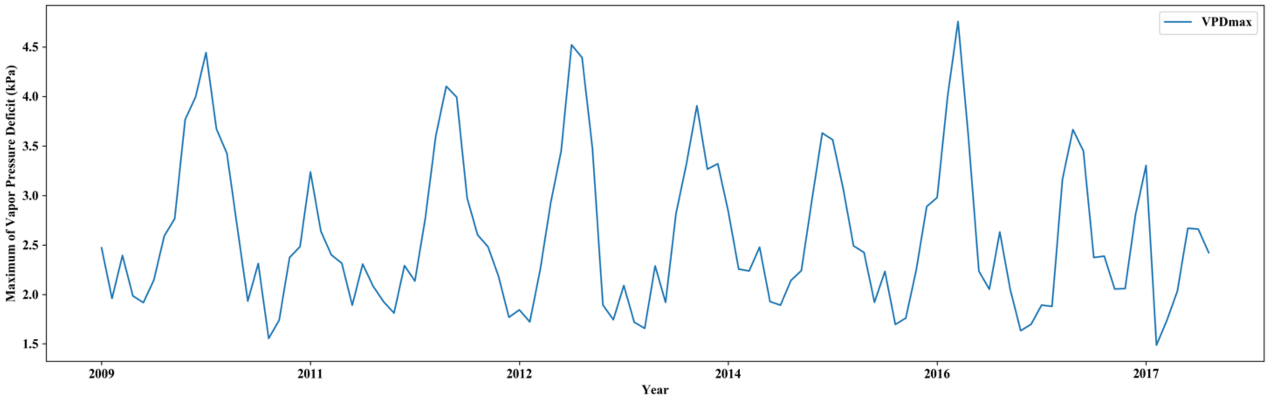

S1 Fig6. Ten years of the monthly maximum of vapor pressure deficit.

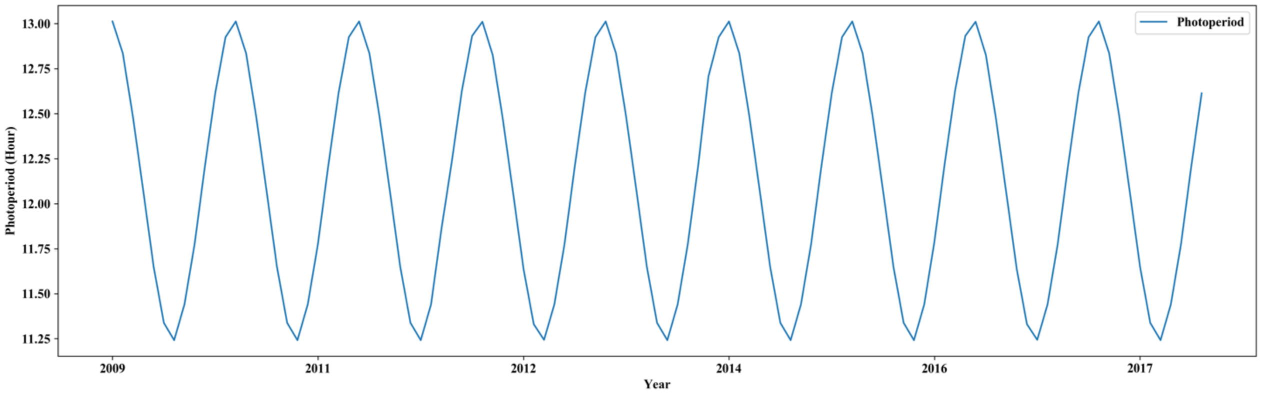

S1 Fig7. Ten years of monthly photoperiod.
