# Supplementary material for: Characterizing and forecasting the responses of tropical forest leaf phenology to El Nino by machine learning algorithms
Source: PLoS One. 2021 Aug 26;16(8):e0255962. doi: 10.1371/journal.pone.0255962 (PMC8389403; doi:10.1371/journal.pone.0255962)
Supplement: S2 Appendix — (PDF) [file pone.0255962.s002.pdf]

S2 Appendix. The detail results of lag time analysis

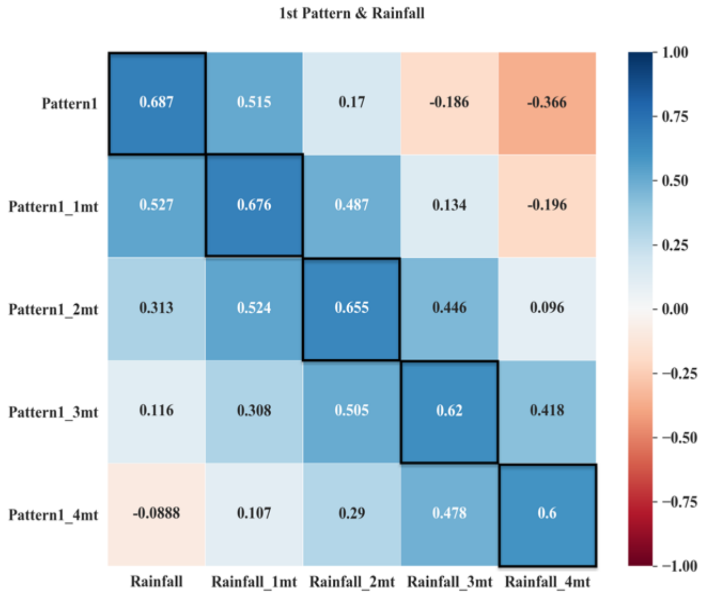

S2 Fig1. The heat map that shows the relationship between the first leave phenology pattern and rainfall.

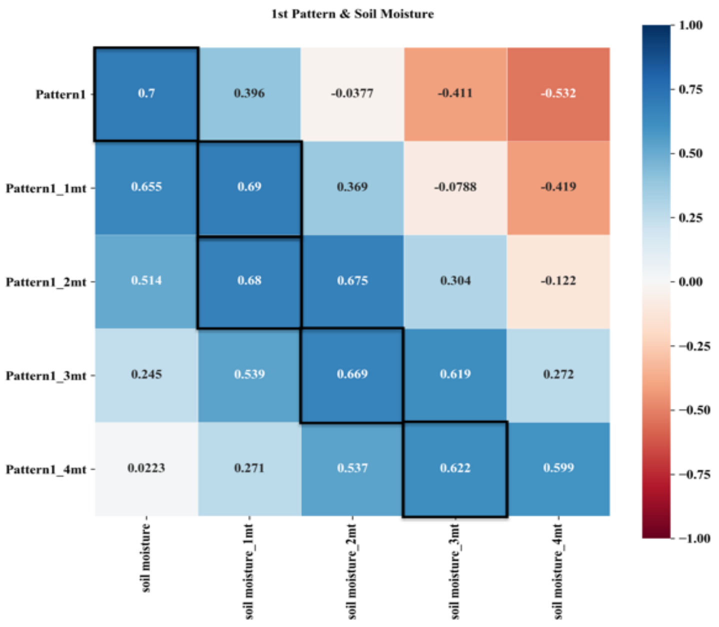

S2 Fig2. The heat map that shows the relationship between the first leave phenology pattern and soil moisture.

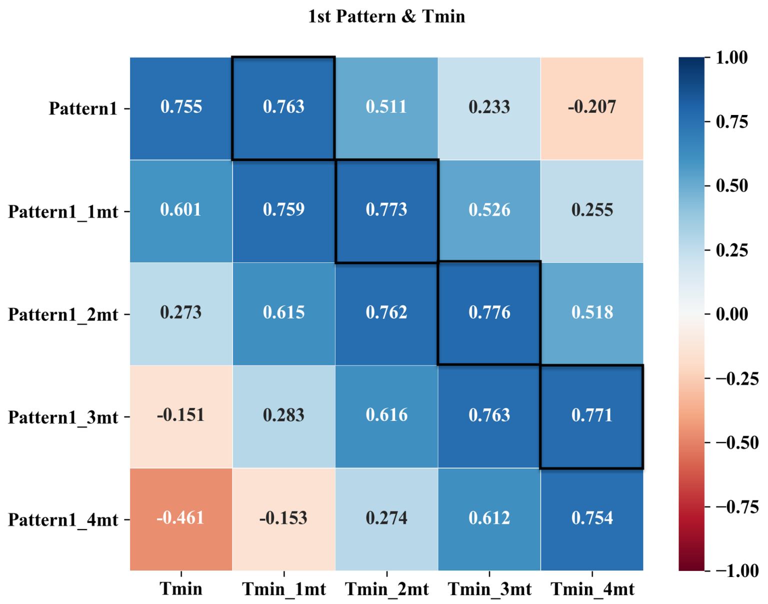

S2 Fig3. The heat map that shows the relationship between the first leave phenology pattern and minimum temperature.

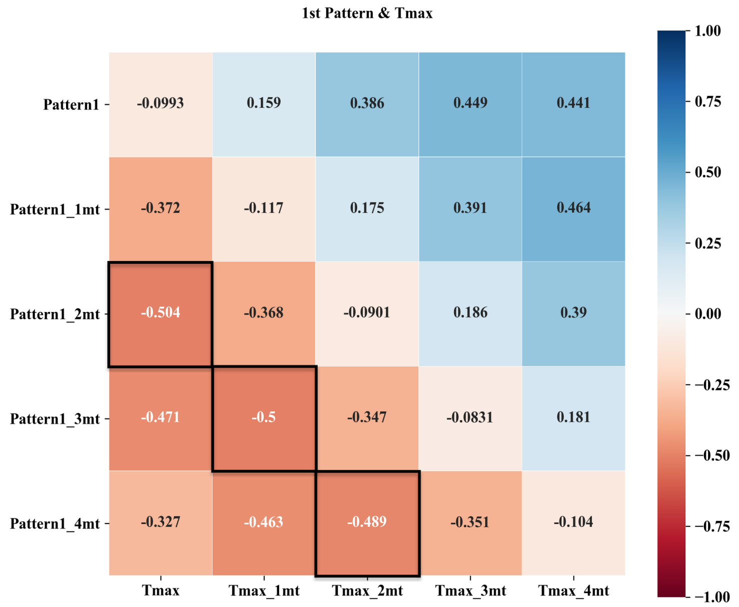

S2 Fig4. The heat map that shows the relationship between the first leave phenology pattern and maximum temperature.

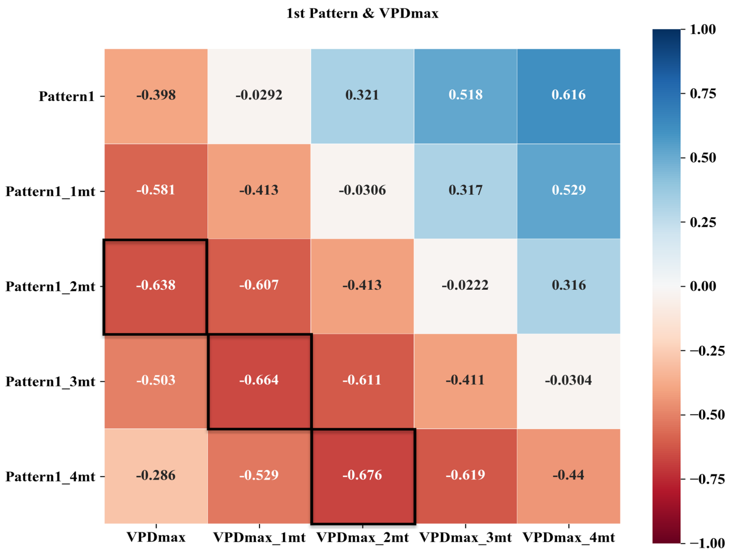

S2 Fig5. The heat map that shows the relationship between the first leave phenology pattern and maximum of vapor pressure deficit.

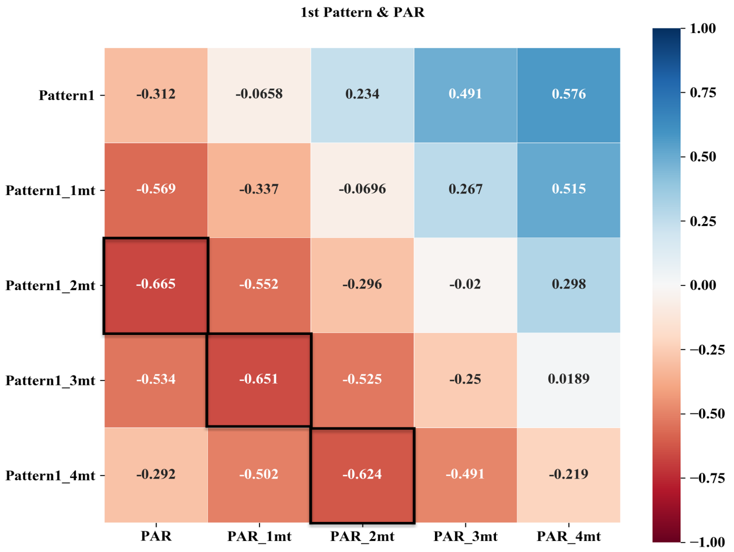

S2 Fig6. The heat map that shows the relationship between the first leave phenology pattern and photosynthetically active radiation.

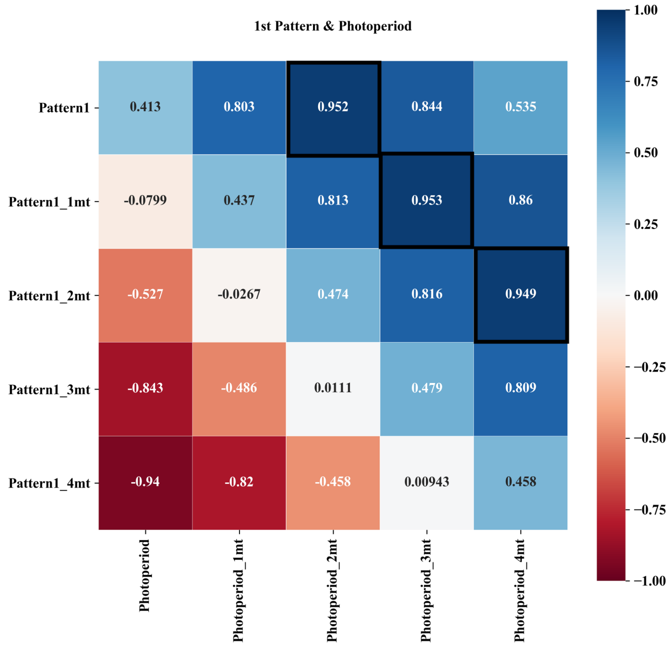

S2 Fig7. The heat map that shows the relationship between the first leave phenology pattern and photoperiod.

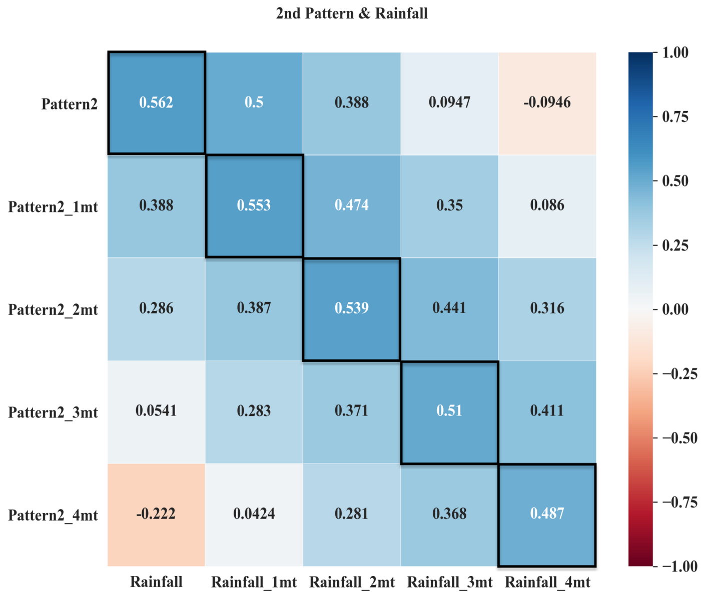

S2 Fig8. The heat map that shows the relationship between the second leave phenology pattern and rainfall.

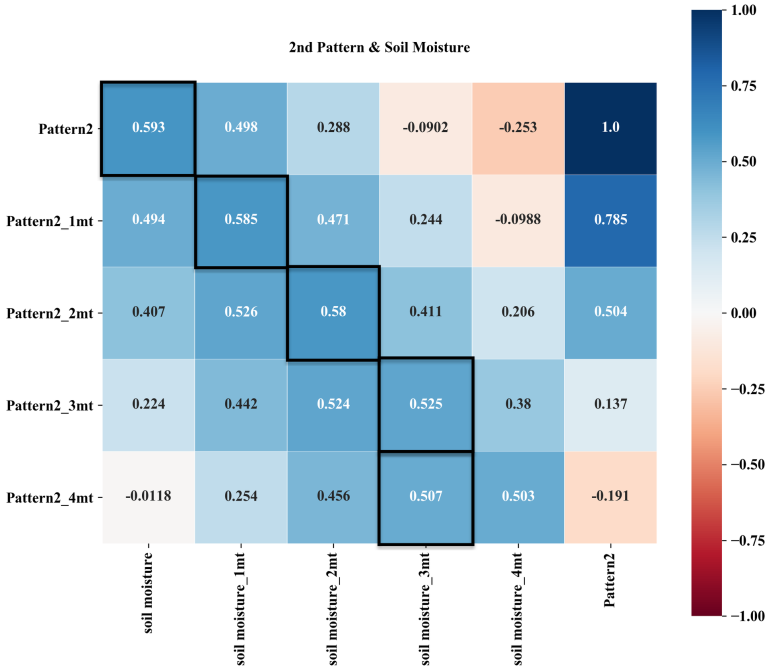

S2 Fig9. The heat map that shows the relationship between the second leave phenology pattern and soil moisture.

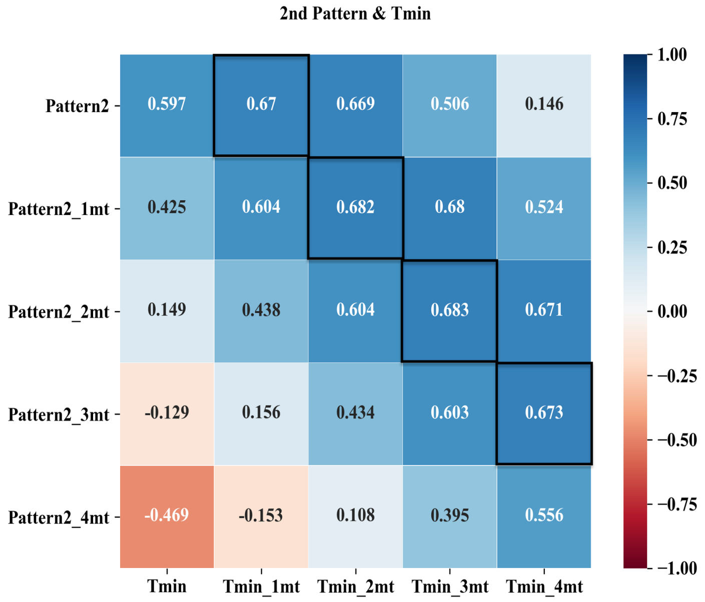

S2 Fig10. The heat map that shows the relationship between the second leave phenology pattern and minimum temperature.

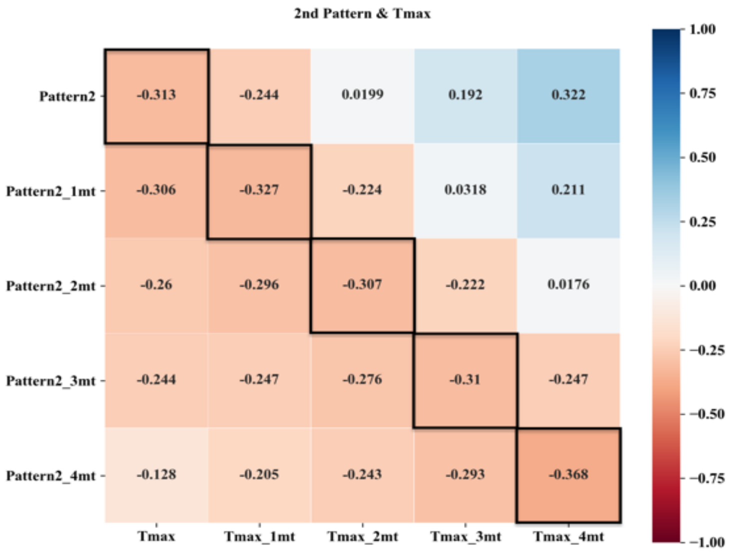

S2 Fig11. The heat map that shows the relationship between the second leave phenology pattern and maximum temperature.

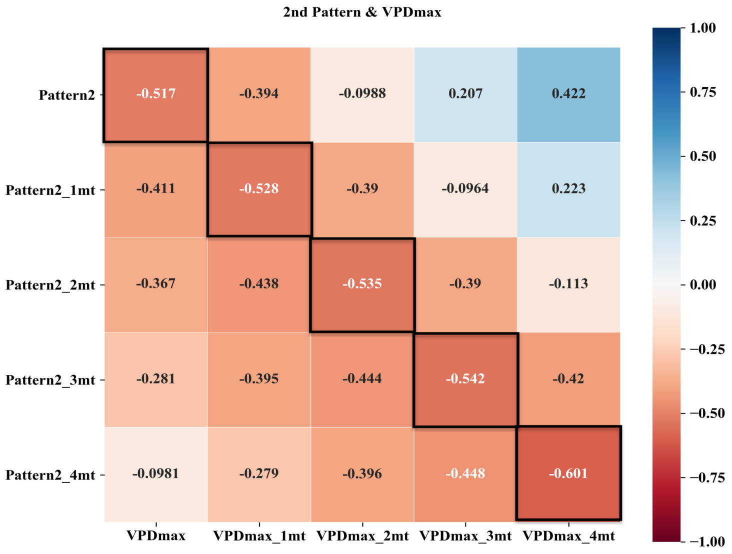

S2 Fig12. The heat map that shows the relationship between the second leave phenology pattern and maximum of vapor pressure deficit.

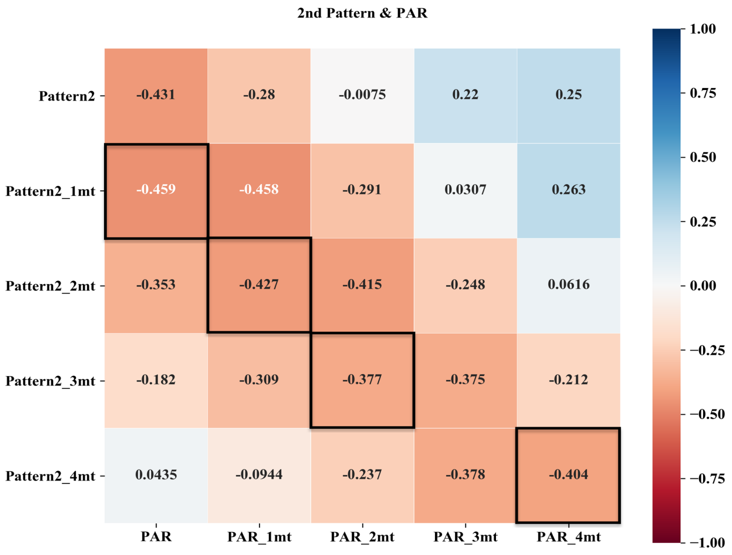

S2 Fig13. The heat map that shows the relationship between the second leave phenology pattern and photosynthetically active radiation.

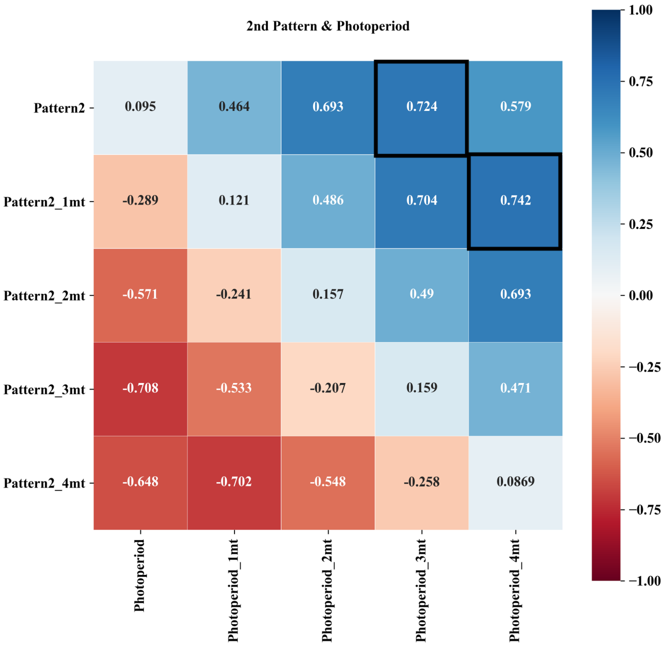

S2 Fig14. The heat map that shows the relationship between the second leave phenology pattern and photoperiod.

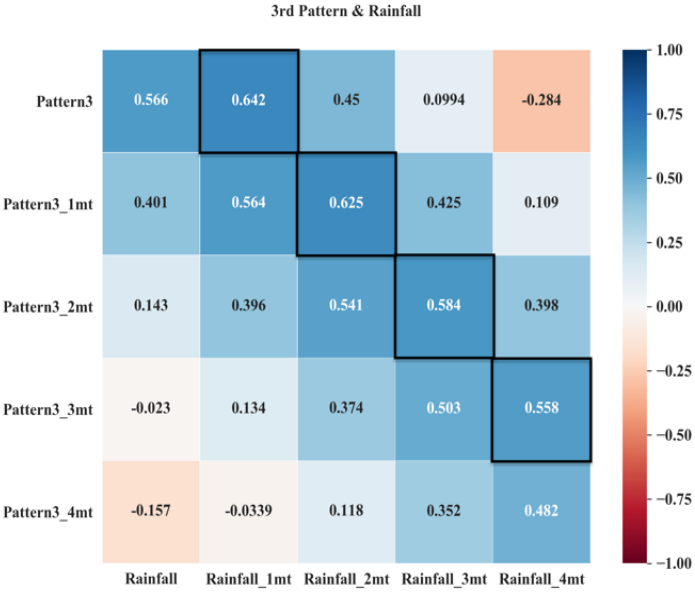

S2 Fig15. The heat map that shows the relationship between the third leave phenology pattern and rainfall.

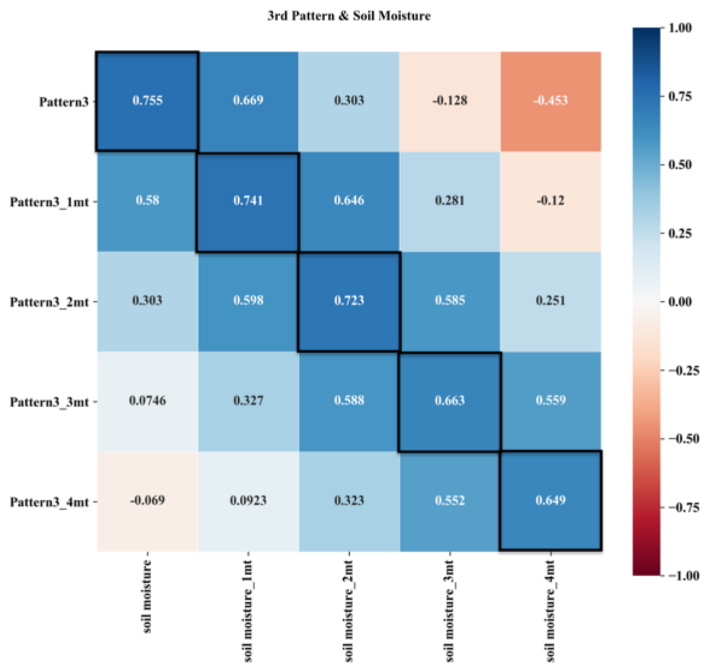

S2 Fig16. The heat map that shows the relationship between the third leave phenology pattern and soil moisture.

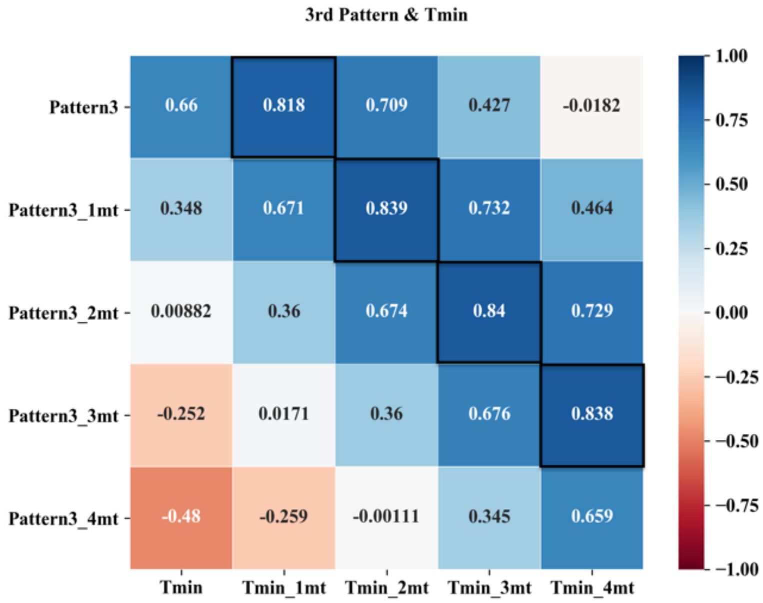

S2 Fig17. The heat map that shows the relationship between the third leave phenology pattern and minimum temperature.

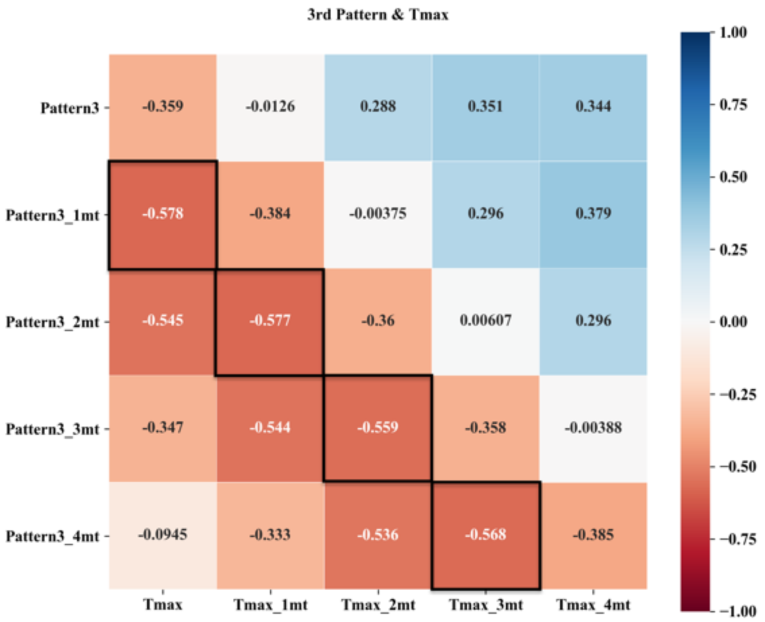

S2 Fig18. The heat map that shows the relationship between the third leave phenology pattern and maximum tenperature.

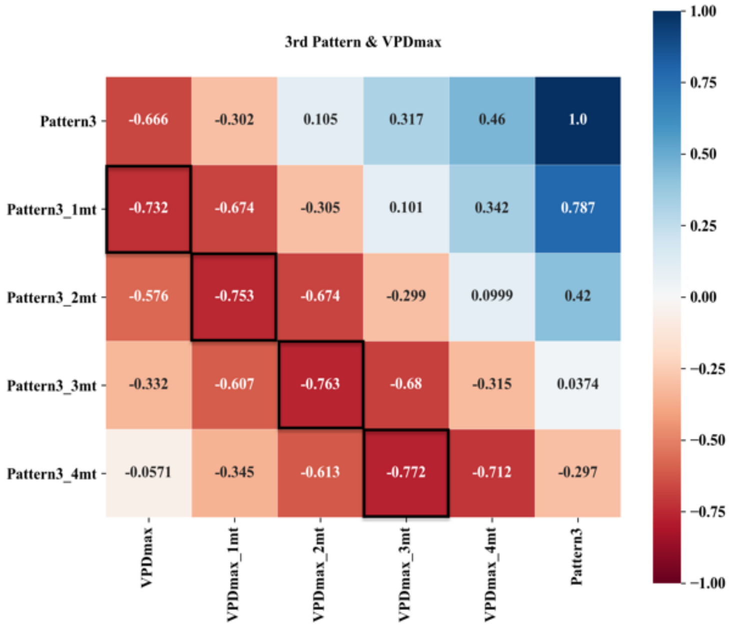

S2 Fig19. The heat map that shows the relationship between the third leave phenology pattern and maximum of vapor pressure deficit.

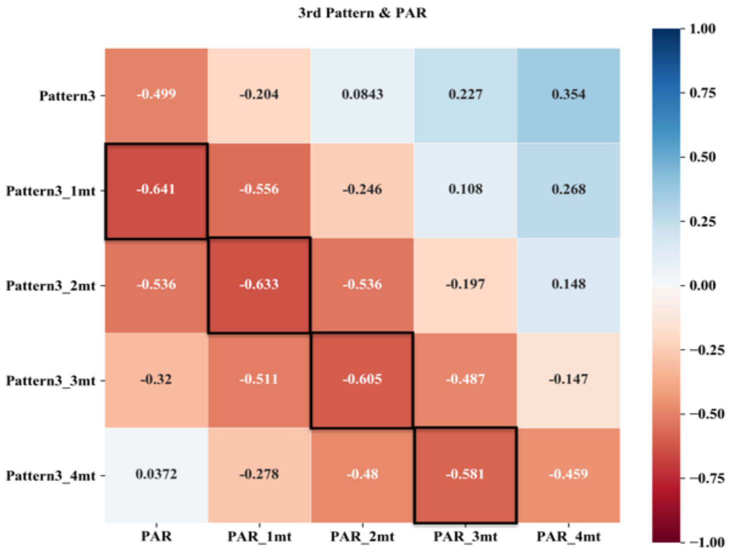

S2 Fig20. The heat map that shows the relationship between the third leave phenology pattern and photosynthetically active radiation.

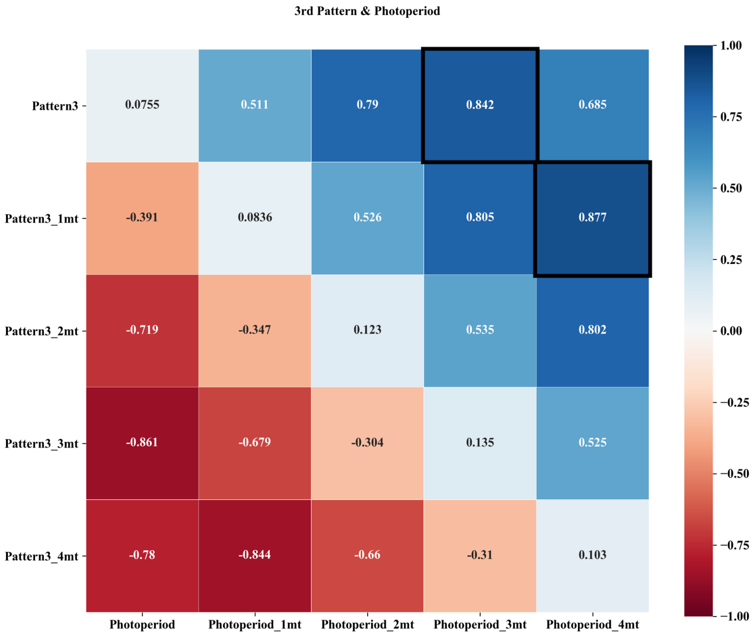

S2 Fig21. The heat map that shows the relationship between the third leave phenology pattern and photoperiod.

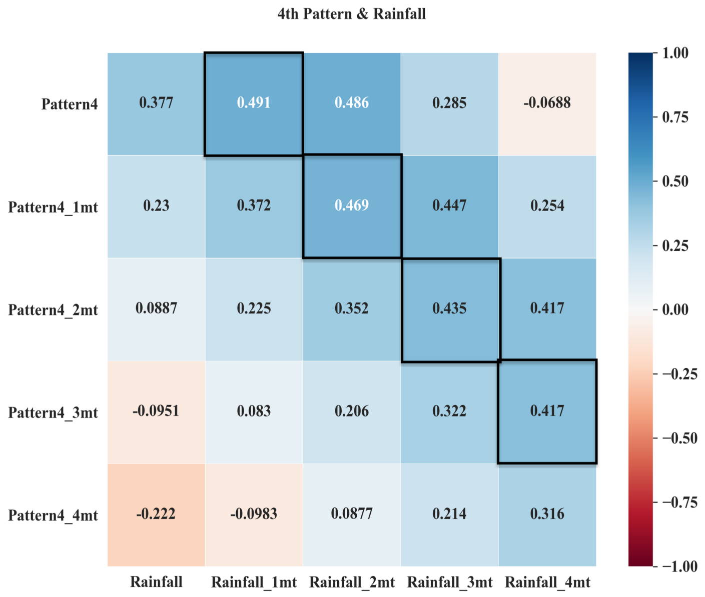

S2 Fig22. The heat map that shows the relationship between the fourth leave phenology pattern and rainfall.

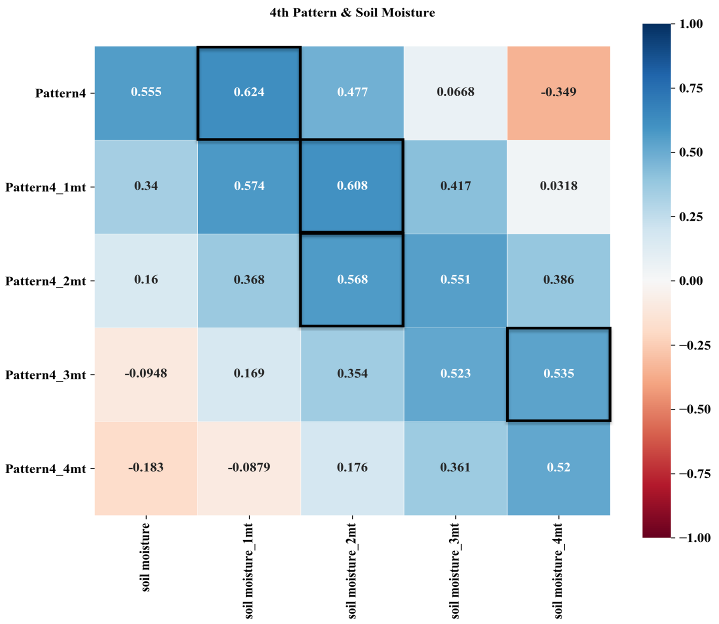

S2 Fig23. The heat map that shows the relationship between the fourth leave phenology pattern and soil moisture.

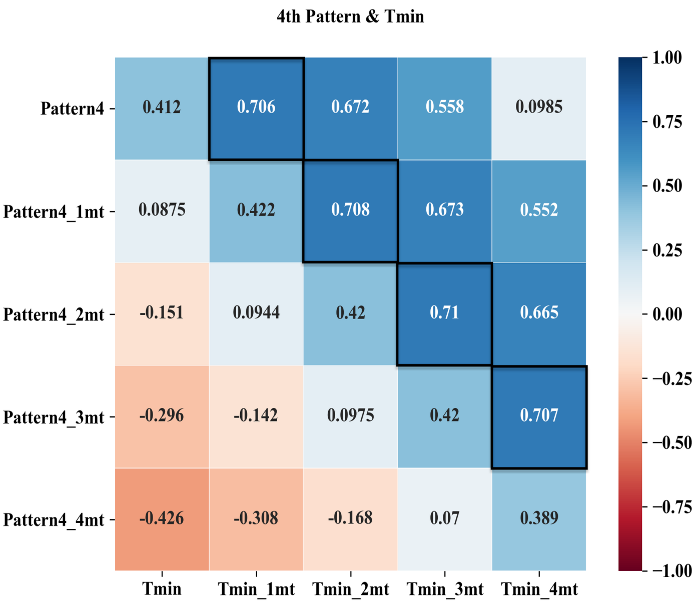

S2 Fig24. The heat map that shows the relationship between the fourth leave phenology pattern and minimum temperature.

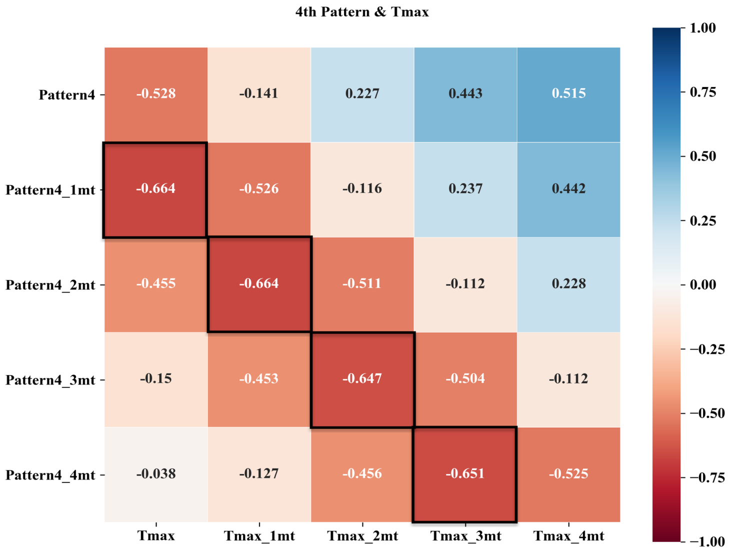

S2 Fig25. The heat map that shows the relationship between the fourth leave phenology pattern and maximum temperature.

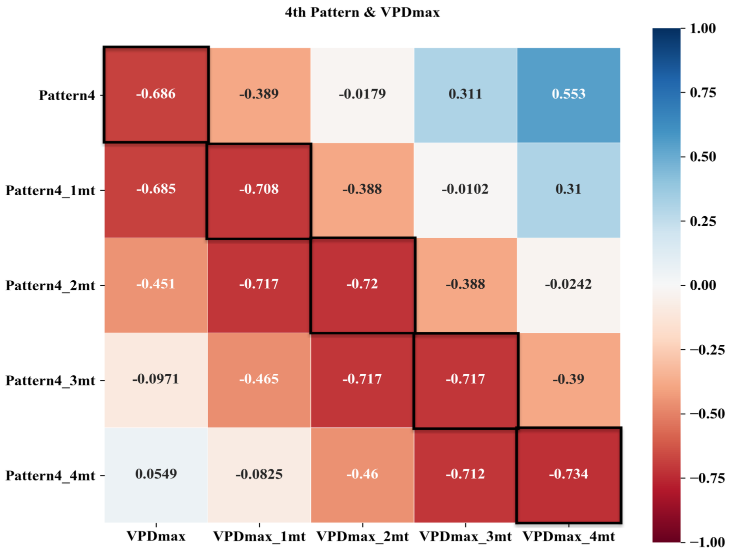

S2 Fig26. The heat map that shows the relationship between the fourth leave phenology pattern and maximum of vapor pressure deficit.

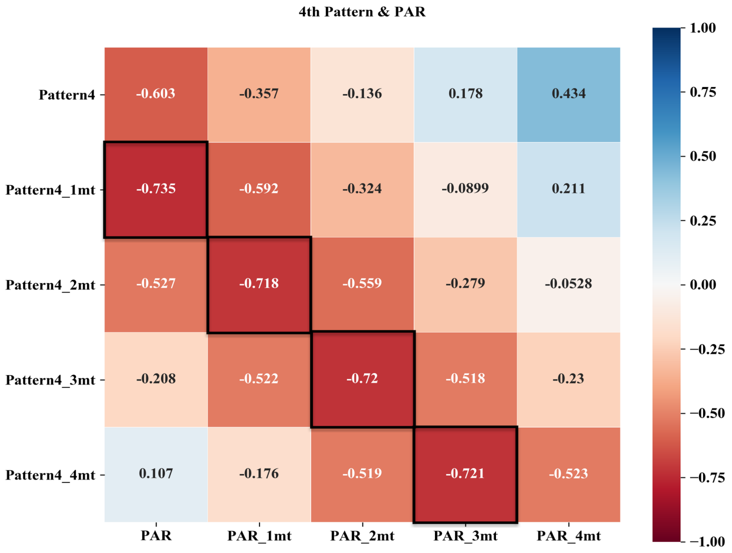

S2 Fig27. The heat map that shows the relationship between the fourth leave phenology pattern and photosynthetically active radiation.

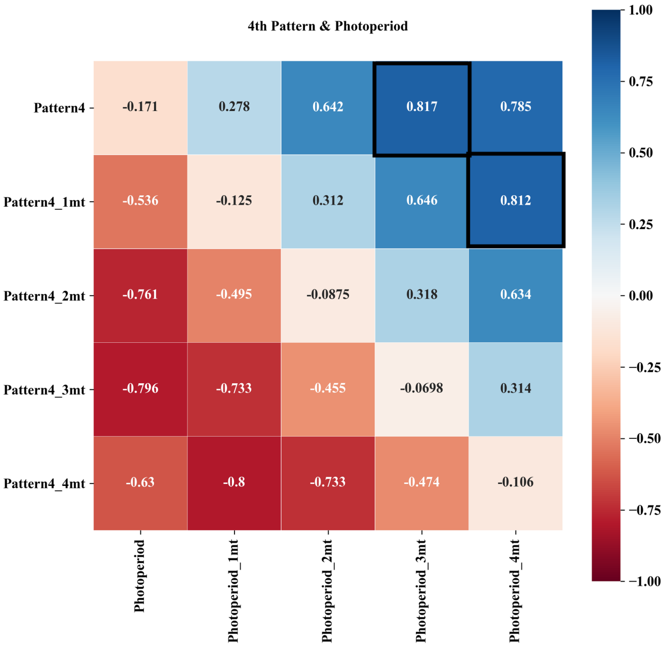

S2 Fig28. The heat map that shows the relationship between the fourth leave phenology pattern and photoperiod.

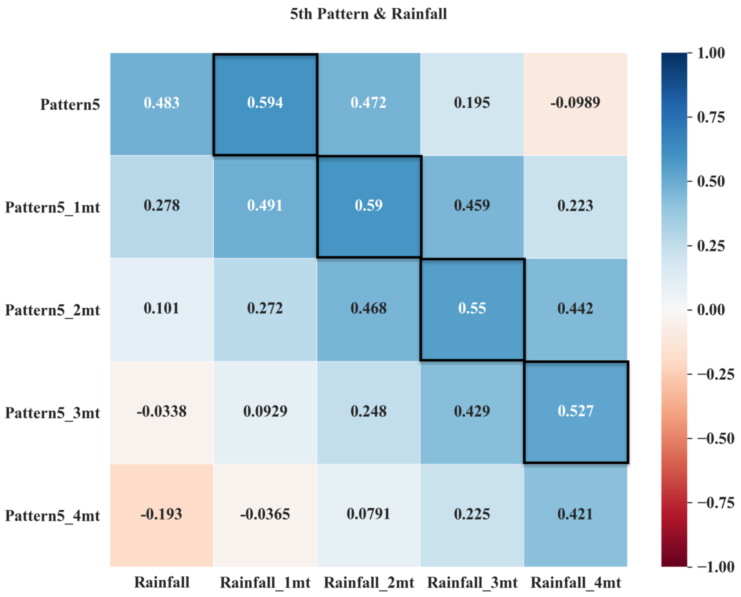

S2 Fig29. The heat map that shows the relationship between the fifth leave phenology pattern and rainfall.

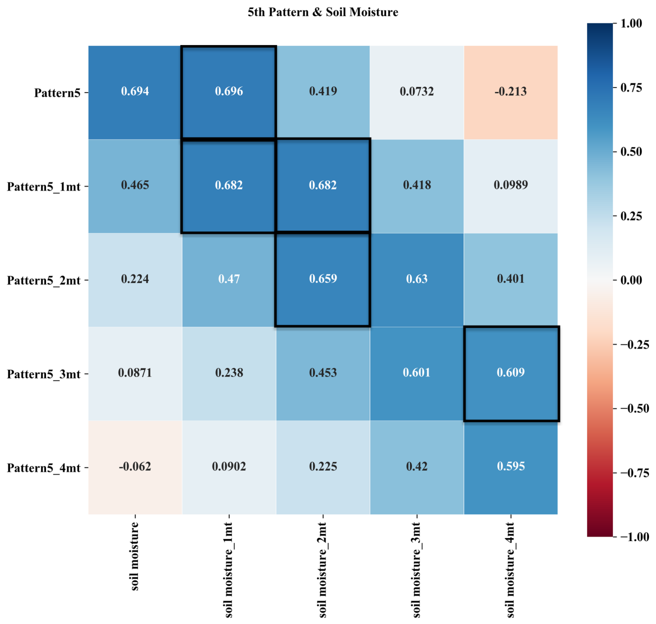

S2 Fig30. The heat map that shows the relationship between the fifth leave phenology pattern and soil moisture.

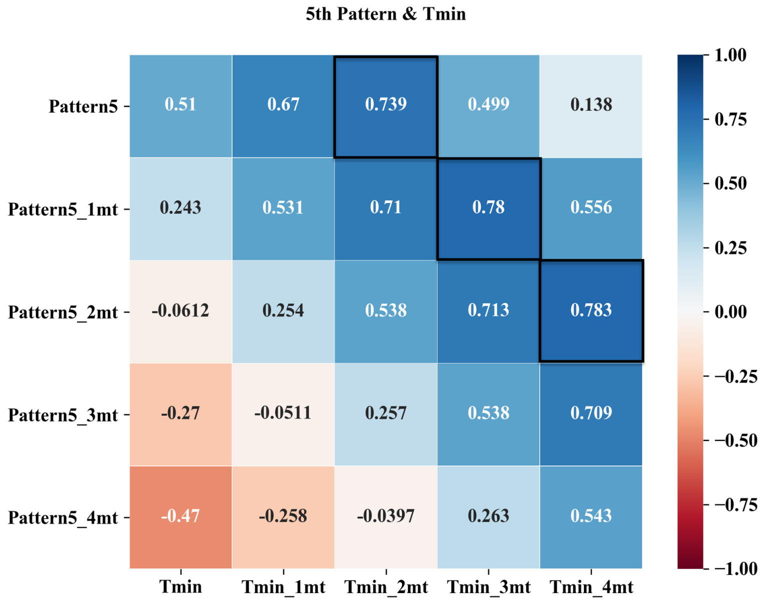

S2 Fig31. The heat map that shows the relationship between the fifth leave phenology pattern and minimum temperature.

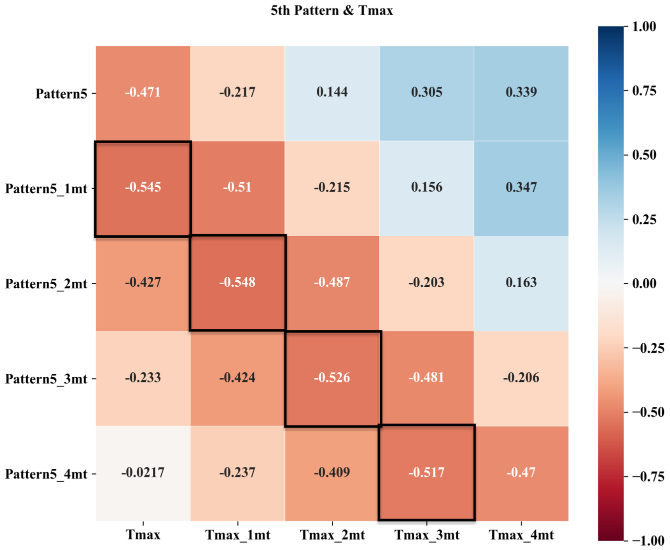

S2 Fig32. The heat map that shows the relationship between the fifth leave phenology pattern and maximum temperature.

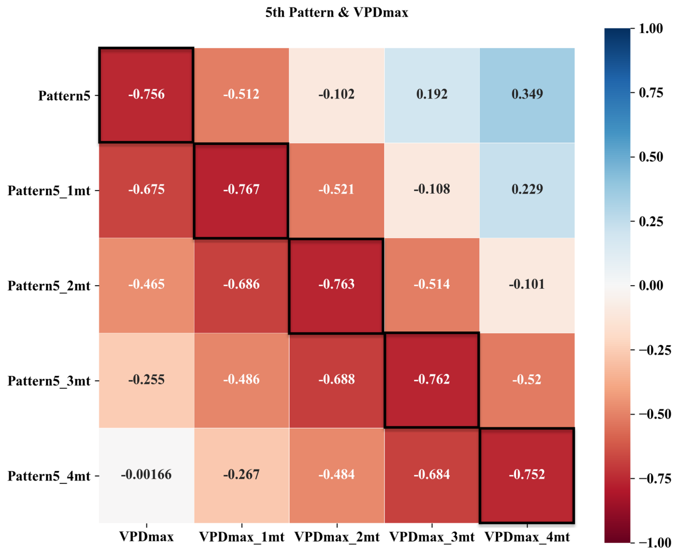

S2 Fig33. The heat map that shows the relationship between the fifth leave phenology pattern and maximum of vapor pressure deficit.

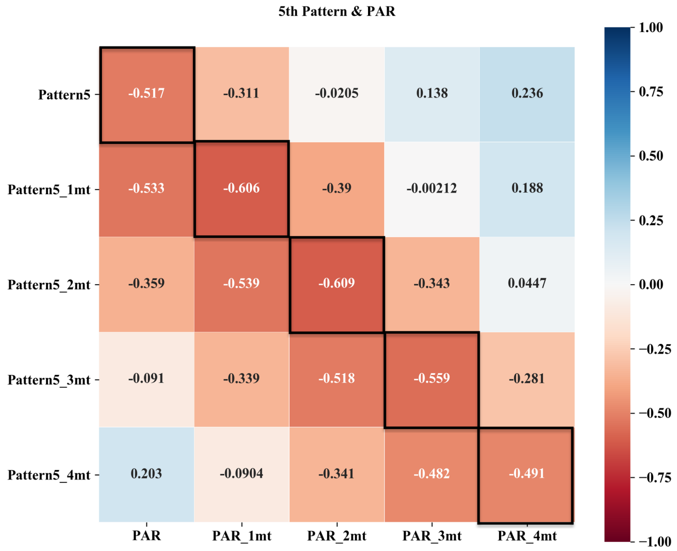

S2 Fig34. The heat map that shows the relationship between the fifth leave phenology pattern and photosynthetically active radiation.

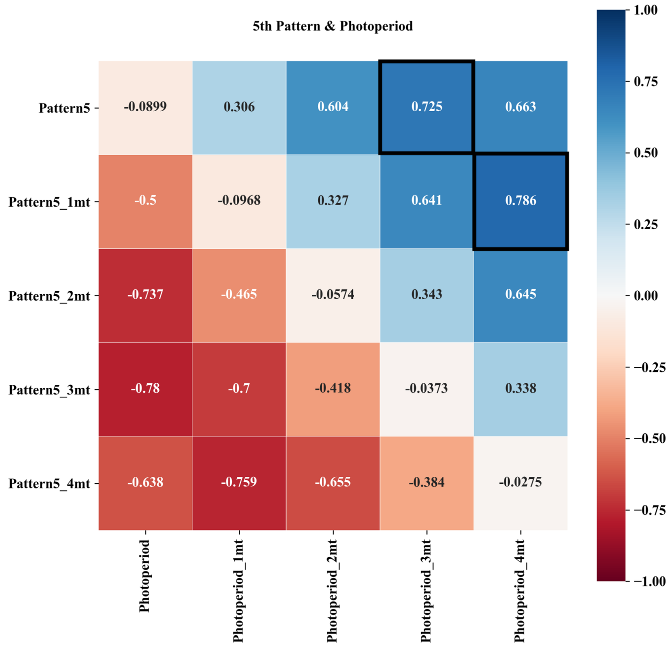

S2 Fig35. The heat map that shows the relationship between the fifth leave phenology pattern and photoperiod.

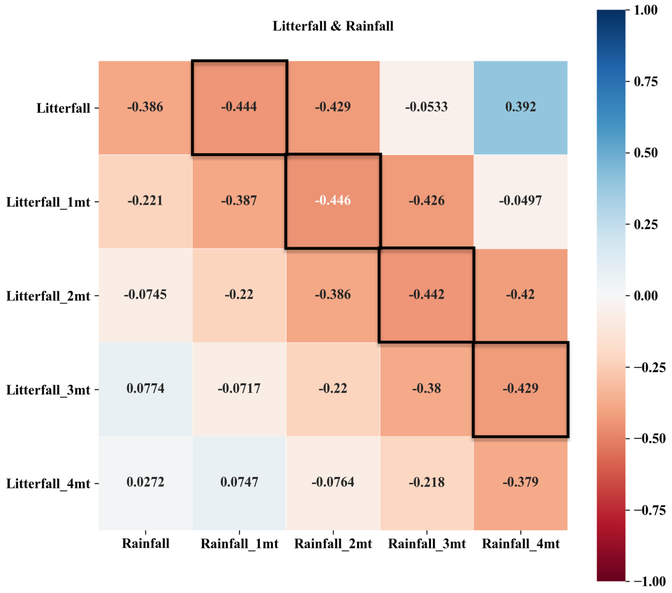

S2 Fig36. The heat map that shows the relationship between the litterfall and rainfall.

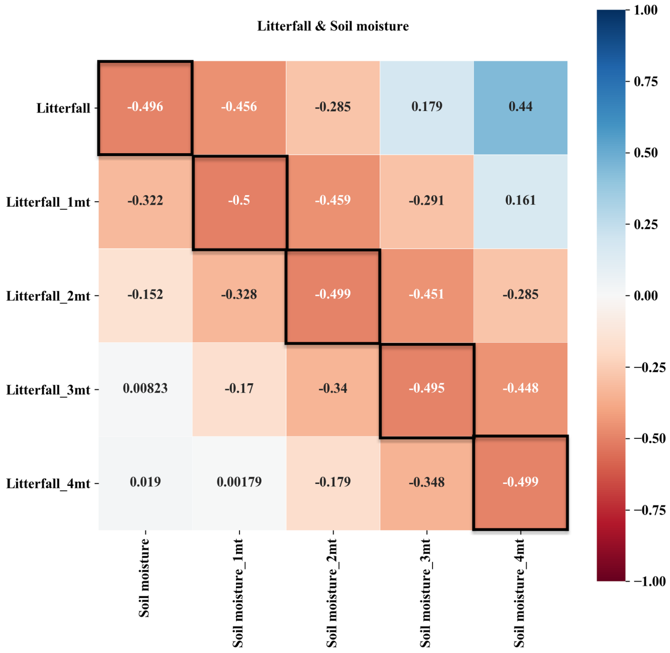

S2 Fig37. The heat map that shows the relationship between the litterfall and soil moisture.

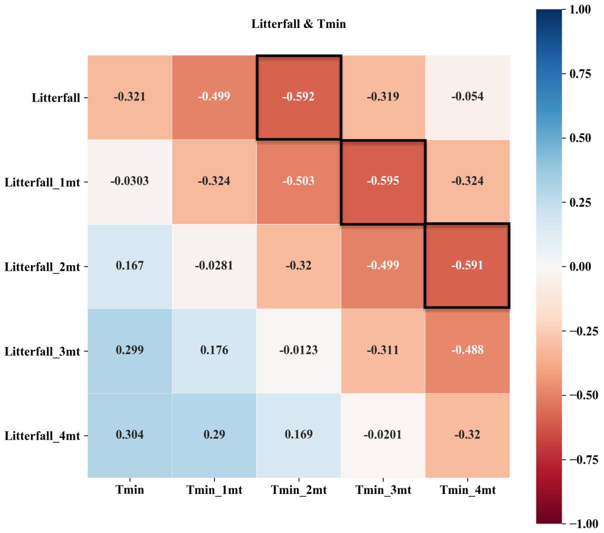

S2 Fig38. The heat map that shows the relationship between the litterfall and minimum temperature.

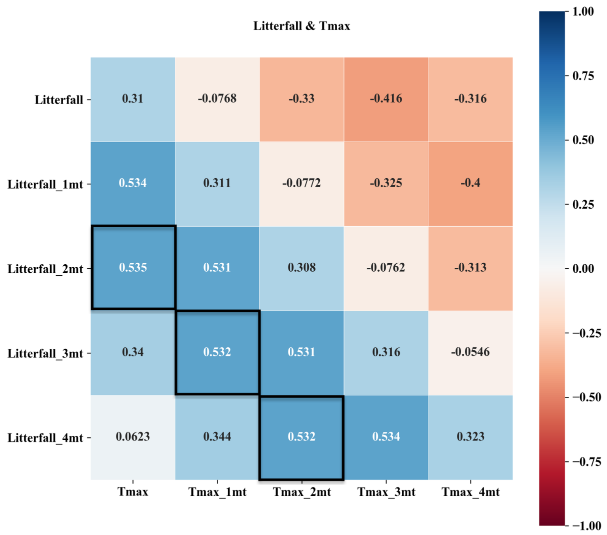

S2 Fig39. The heat map that shows the relationship between the litterfall and maximum temperature.

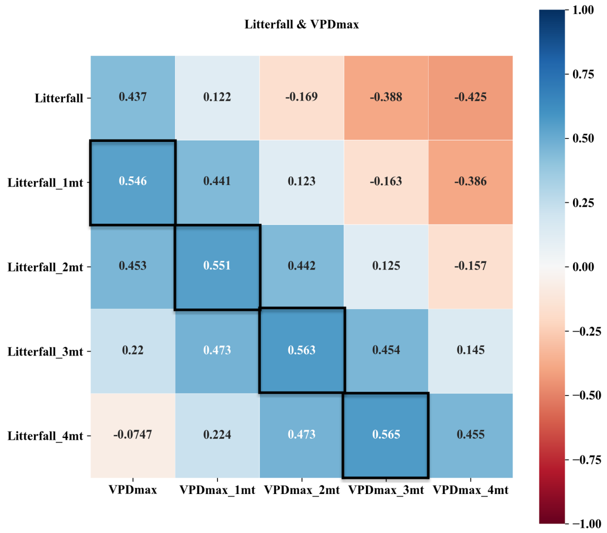

S2 Fig40. The heat map that shows the relationship between the litterfall and maximum of vapor pressure deficit.

.

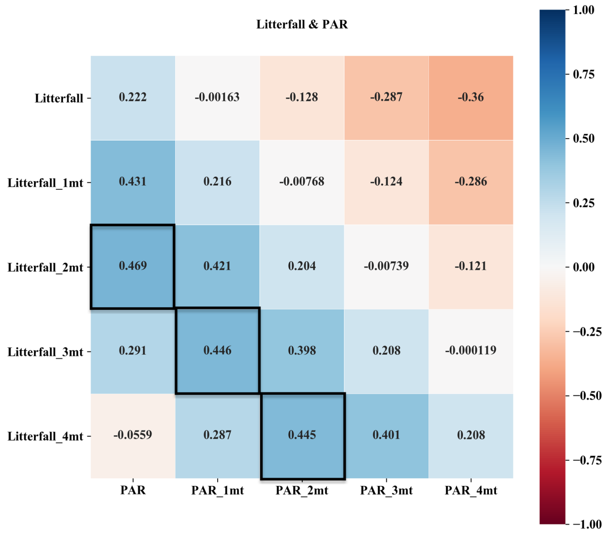

S2 Fig41. The heat map that shows the relationship between the litterfall and photosynthetically active radiation.

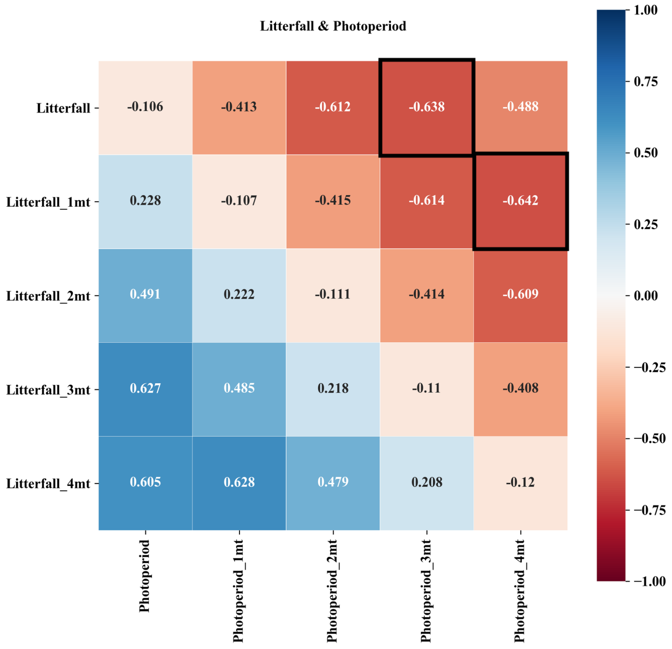

S2 Fig42. The heat map that shows the relationship between the litterfall and photoperiod.
